# First Glimpse of Gut Microbiota of Quarantine Insects in China

**DOI:** 10.1016/j.gpb.2022.04.005

**Published:** 2022-05-24

**Authors:** Yanxue Yu, Qi Wang, Ping Zhou, Na Lv, Wei Li, Fangqing Zhao, Shuifang Zhu, Di Liu

**Affiliations:** 1 Institute of Plant Quarantine, Chinese Academy of Inspection and Quarantine, Beijing 100176, China; 2 Institute of Physical Science and Information Technology, Anhui University, Hefei 230601, China; 3 Computational Virology Group, Center for Bacteria and Virus Resources and Bioinformation, Wuhan Institute of Virology, Chinese Academy of Sciences, Wuhan 430071, China; 4 CAS Key Laboratory of Pathogenic Microbiology and Immunology, Institute of Microbiology, Chinese Academy of Sciences, Beijing 100101, China; 5 Computational Genomics Lab, Beijing Institutes of Life Science, Chinese Academy of Sciences, Beijing 102206, China; 6 University of Chinese Academy of Sciences, Beijing 100049, China

**Keywords:** Quarantine insect, Geographical origin, 16S rRNA, Gut microbiota, Genus level

## Abstract

**Quarantine insects** are economically important pests that frequently invade new habitats. A rapid and accurate monitoring method to trace the geographical sources of invaders is required for their prevention, detection, and eradication. Current methods based on genetics are typically time-consuming. Here, we developed a novel tracing method based on insect **gut microbiota**. The source location of the insect gut microbiota can be used to rapidly determine the **geographical origin** of the insect. We analyzed 179 gut microbiota samples from 591 individuals of 22 quarantine insect species collected from 36 regions in China. The gut microbiota of these insects primarily included Actinobacteria, Bacteroidetes, Cyanobacteria, Firmicutes, Proteobacteria, and Tenericutes. The diversity of the insect gut microbiota was closely associated with geographical and environmental factors. Different insect species could be distinguished based on the composition of gut microbiota at the phylum level. Populations of individual insect species from different regions could be distinguished based on the composition of gut microbiota at the phylum, class, and order levels. A method for determining the geographical origins of invasive insect species has been established; however, its practical application requires further investigations before implementation.

## Introduction

Insects are common, diverse, and widely distributed [1]. Quarantine insects are species that have been introduced and are damaging to agriculture, forestry, stored products, and human health. Countries or regions must take preventive and control measures to reduce the introduction of and damage caused by quarantine insects. Quarantine insects cause annual global losses of billions of dollars [2], [3], and China is severely affected by numerous species [4], [5]. There are 150 species and genera of quarantine insects on the List of Imported Plant Quarantine Pests in the People’s Republic of China (https://www.zys.moa.gov.cn/flfg/201904/t20190428_6245344.htm). Quarantine insects exhibit a relatively limited distribution. For example, *Leptinotarsa decemlineata* is primarily distributed in the northeast and northwest of China, whereas *Lissorhoptrus oryzophilus* is widely distributed in areas where its host plant (*Oryza sativa*) is grown.

Rapid and accurate methods are required to identify geographical sources of quarantine insects. This would aid in their detection, monitoring, prevention, and eradication. Current methods primarily use genetic tests such as DNA barcode technology [6], [7], restriction fragment length polymorphisms (RFLPs) [8], single nucleotide polymorphisms (SNPs) [9], and microsatellite markers [10]. These methods are based on gene flow between populations and require several insect generations for completion. Genetic changes in populations from different areas can only be used to infer movement routes [5]. No methods can trace the origin of an individual insect within a short interval.

The gut microbiota genome is sometimes considered to be the second genome of an insect species. Gut microbiota is closely associated with the environment of the insect. Thus, it is variable and can change rapidly. Microbe characteristics are often related to their areas of origin. Specific microbes can interact with quarantine insects through their diet and contribute to their gut microbiota. Gut microbiota may provide clues to the geographical source of a quarantine insect. The 16S rRNA gene sequencing technology can be used to detect and identify insect gut microbiota [11]. In this study, we developed a method to determine the geographical sources of quarantine insects using gut microbiota identified via 16S rRNA gene sequencing.

## Results

### Widespread collection of insect samples in China and establishment of DNA extraction method

**Figure 1:**
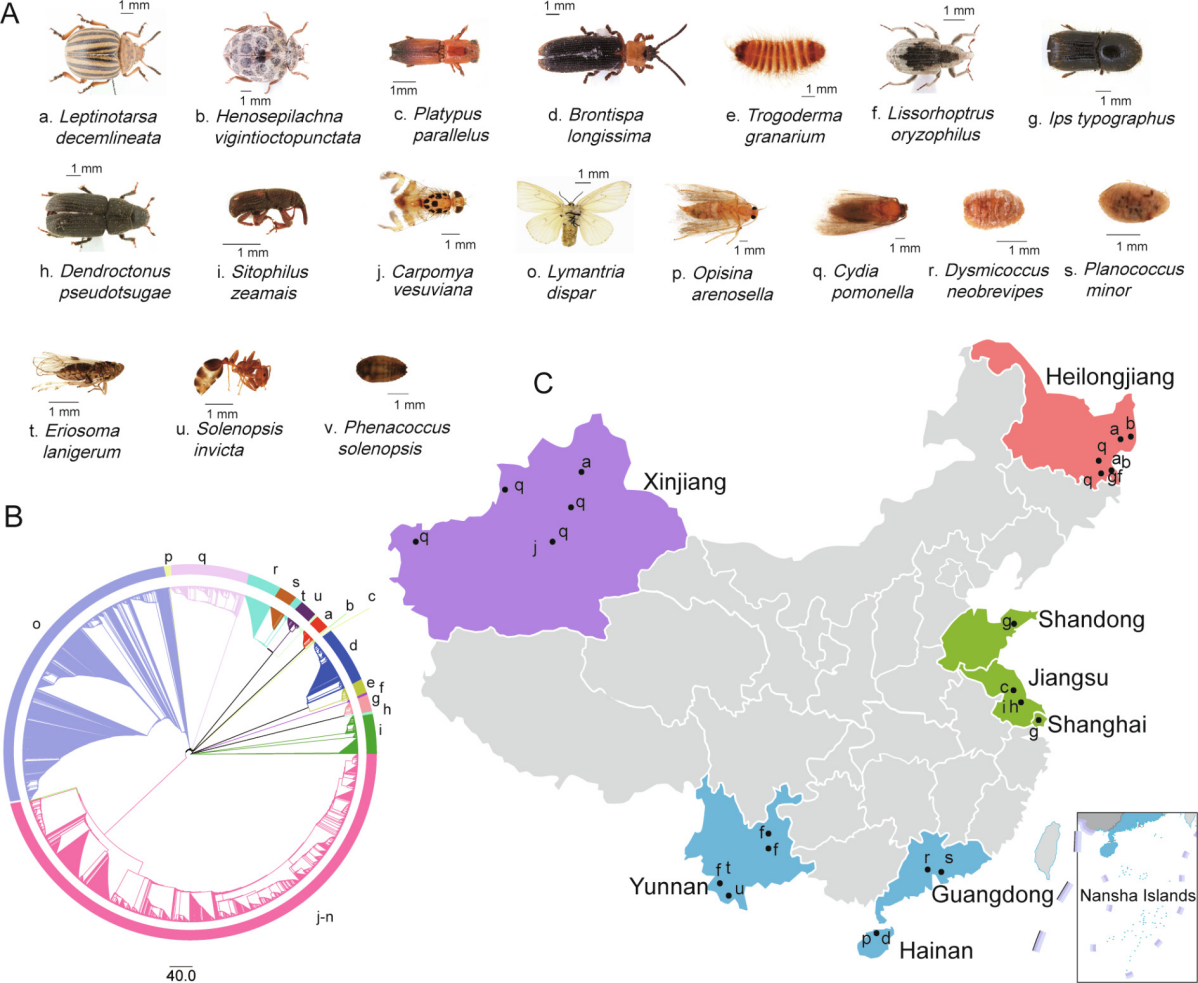
**Collection of insect samples and analysis of evolutionary relationships** **A.** Representative photographs of sampled insects. Lowercase letters represent species corresponding to those shown in (B) and (C). As four species, *Bactrocera correcta* (k), *Bactrocera cucurbitae* (l), *Bactrocera dorsalis* (m), and *Bactrocera tau* (n), have similar morphology to *Carpomya vesuviana* (j), their photographs are not shown. **B.** Phylogenetic tree of insects using the *COI* gene. Each color represents a different species. **C.** Insect sampling sites. Sampling sites are indicated on the map of China, and the four main collection areas are marked with different colors. *COI*, cytochrome oxidase subunit I.

A total of 591 individuals from 22 quarantine insect species belonging to 19 genera and 13 families were collected in China, including 35 individuals that originated outside China and were alive when they were intercepted at the entry ports (Table S1). The representative photographs and the sizes of the species are shown in Figure 1A. Species classification for all insects was performed based on the mitochondrial cytochrome oxidase subunit I (*COI*) gene (Figure 1B). The 36 collection sites were located in eight provinces and spanned the northeast, central, south, and northwest of China (Figure 1C). The factors that influence the gut microbiota of 22 species are listed in Table S1. Among the quarantine insects studied, *Ips typographus* is widely distributed in China and has been found in more than 20 provinces. The distributions of the other 21 species varied, and each was found only in a few provinces. Generally, these insects are widely distributed and established in China.

The sizes of the 591 insect species varied greatly. To ensure that the DNA extraction was not affected by insect size, all insect species were divided into three groups according to their body length. If the body length of adult insects was >5 mm, such as *L*. *decemlineata*, *I*. *typographus*, and *Lymantria dispar*, one individual was used as a sample for gut microbiota analysis. If the body length ranged from 2 mm to 5 mm, such as *L. oryzophilus*, *C. pomonella*, and *Bactrocera cucurbitae*, five individuals collected from one site were used as one sample for DNA extraction. If the body length was <2 mm, such as *Trogoderma granarium*, *Eriosoma lanigerum*, and *Planococcus minor*, 10 individuals collected from one site were used as one sample for DNA extraction. The purpose of several individuals making up a sample was to extract DNA more effectively and reduce individual differences and human impact. A total of 179 gut microbiota samples were obtained from the collected quarantine insects and subjected to 16S rRNA gene sequencing (Table S2).

### Differential composition of gut microbiota at the phylum level could distinguish different quarantine insects

**Figure 2:**
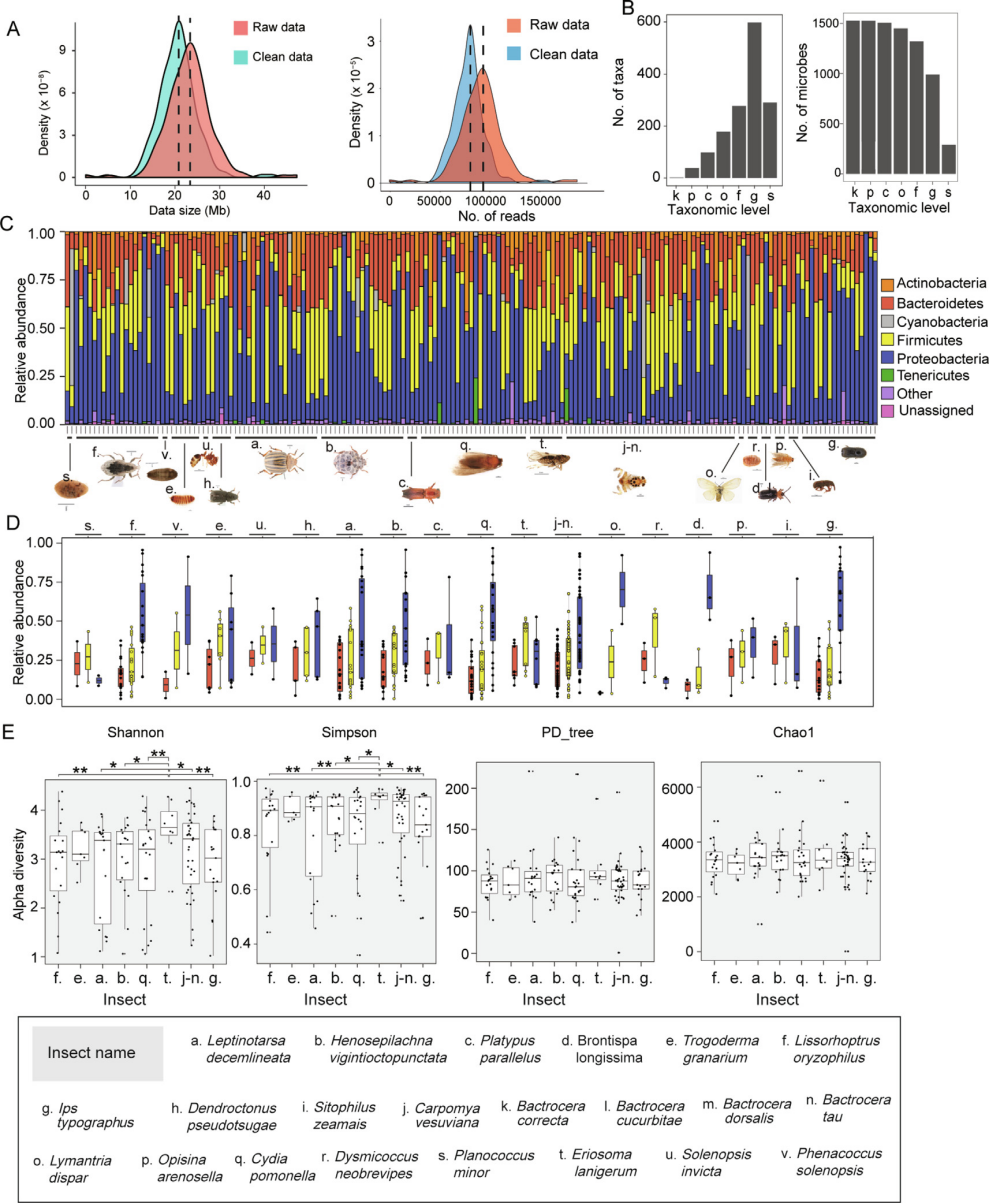
**Depth and breadth of sequencing provide a reliable basis for geographical traceability using gut microbiota** **A.** Data size per sample (left) and sequence number per sample (right). **B.** Number of taxa identified at each taxonomic level (left) and number of microbes identified at each taxonomic level (right). k, kingdom; p, phylum; c, class; o, order; f, family; g, genus; s, species. **C.** Composition of gut microbiota for each sample at the phylum level. **D.** Relative abundance of three dominant phyla in each insect species. The colors of the box correspond to those shown in (C). **E.** Alpha diversity of eight representative insect species (*, *P* < 0.05; **, *P* < 0.01).

All 179 samples were sequenced simultaneously, producing 16,465,986 reads. Each sample contained 50,000–150,000 reads and produced 15–37 Mb of data (Figure 2A). After quality control, 50,000–125,000 reads with 15–32 Mb remained for each sample (Figure 2A). Clean data were subsequently classified using QIIME software [12]. We identified a total of 1527 microbes at various taxonomic levels, with all 1527 microbes belonging to two kingdoms (archaea and bacteria), 1525 microbes to 38 phyla, 1505 microbes to 98 classes, 1450 microbes to 178 orders, 1320 microbes to 277 families, 989 microbes to 598 genera, and 290 microbes to 290 species (Figure 2B).

In view of the complexity of the data, we analyzed the composition of each gut microbiota sample at the phylum level. As shown in Figure 2C, Bacteroidetes, Firmicutes, and Proteobacteria were the three most dominant phyla in each sample; Actinobacteria was ubiquitous; and Cyanobacteria and Tenericutes were detected in some of the samples. The proportions of these phyla were different in each gut microbiota sample because of different insect host species and collection sites. Proteobacteria was the dominant phylum with the highest proportion, followed by Firmicutes and Bacteroidetes (Figure 2D). Furthermore, eight insect species with a sample number >5 were subjected to alpha diversity analysis. Except for *E*. *lanigerum,* all seven other species exhibited no significant differences in Shannon, Simpson, PD_tree, or Chao1 indices (Figure 2E). However, the Shannon and Simpson indices of *E*. *lanigerum* showed significant differences from those of other insect species, except *Trogoderma granarium* (Figure 2E).

### Linear discriminant analysis is more suitable than principal component analysis for distinguishing quarantine insects by their geographical sources

**Figure 3:**
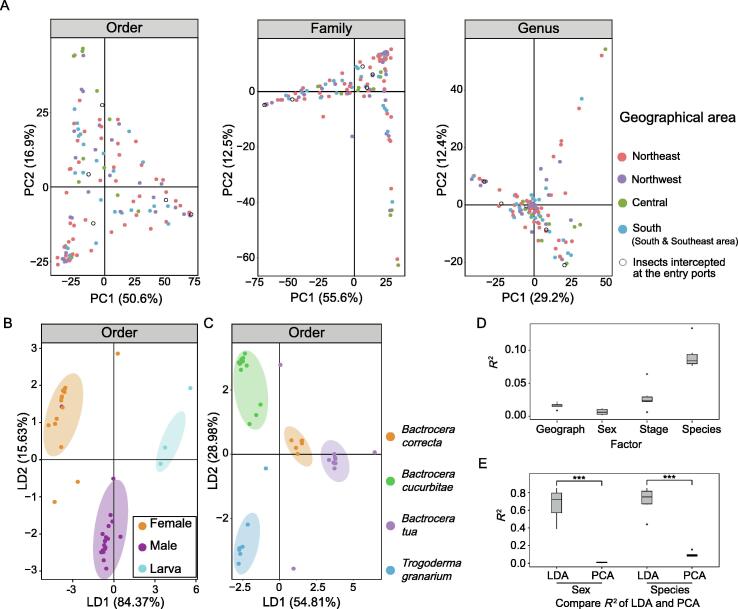
**Comparison of PCA and LDA performances for distinguishing quarantine insects by different factors** **A.** Performance of PCA method for distinguishing insects considering the geographical factor. Dots in different colors represent insect samples collected from five different geographical areas of China (as shown in Figure 1C), and the hollow circle indicates the insect samples intercepted at the entry ports. **B.** Performance of LDA method for distinguishing insects considering the sex factor. **C.** LDA method for distinguishing insects considering the insect species factor. **D.** Effect sizes of factors affecting the gut microbiota of insects. *R*^2^ was calculated by the ADONIS test. **E.** Comparison of ADONIS effect sizes between LDA and PCA. **, *P* < 0.01; ***, *P* < 0.001 (*t*-test). PCA, principal component analysis; LDA, linear discriminant analysis.

To link the geographical sources and the gut microbiota of quarantine insects, we analyzed the microbiota data of all insects collected from the field using principal component analysis (PCA). However, the dots representing insects collected from the five geographical areas of China overlapped (Figure 3A, Figure S1). A previous study has shown that multiple factors affect the gut microbiota of insects, including insect species, developmental stage, diet, sex, and geographical location [13], [14]. According to the ADONIS test results based on the normalized abundance of gut microbiota, the effect size of the geographical factor (*R*^2^ = 0.016) was higher than that of the sex factor (*R*^2^ = 0.007) yet lower than that of the developmental stage (*R*^2^ = 0.028) and insect species (*R*^2^ = 0.09). This suggests that the geographical source of the insects was not the dominant factor affecting gut microbiota. Therefore, we performed linear discriminant analysis (LDA), which is a classification algorithm based on prior information. We compared the performances of PCA and LDA using the ADONIS test for reared insects whose geographical sources and diet factors were controlled. Given sex as prior information, LDA (*R*^2^ = 0.67, *P* = 0.001) was able to better discriminate insects than PCA (*R*^2^ = 0.01, *P* = 0.881) (Figure 3B, Figures S2A and S3A). Given insect species as prior information, LDA was also better than PCA for discriminating insects (*R*^2^ = 0.71, *P* = 0.001 for LDA; *R*^2^ = 0.10, *P* = 0.36 for PCA; Figure 3C, Figures S2B and S3B). These results suggest that LDA has a better diagnostic ability than PCA for extracting specific factors affecting gut microbiota (Figure 3D and E).

### Class and order are proper taxonomic levels of gut microbiota to distinguish the geographical clusters of quarantine insects

**Figure 4:**
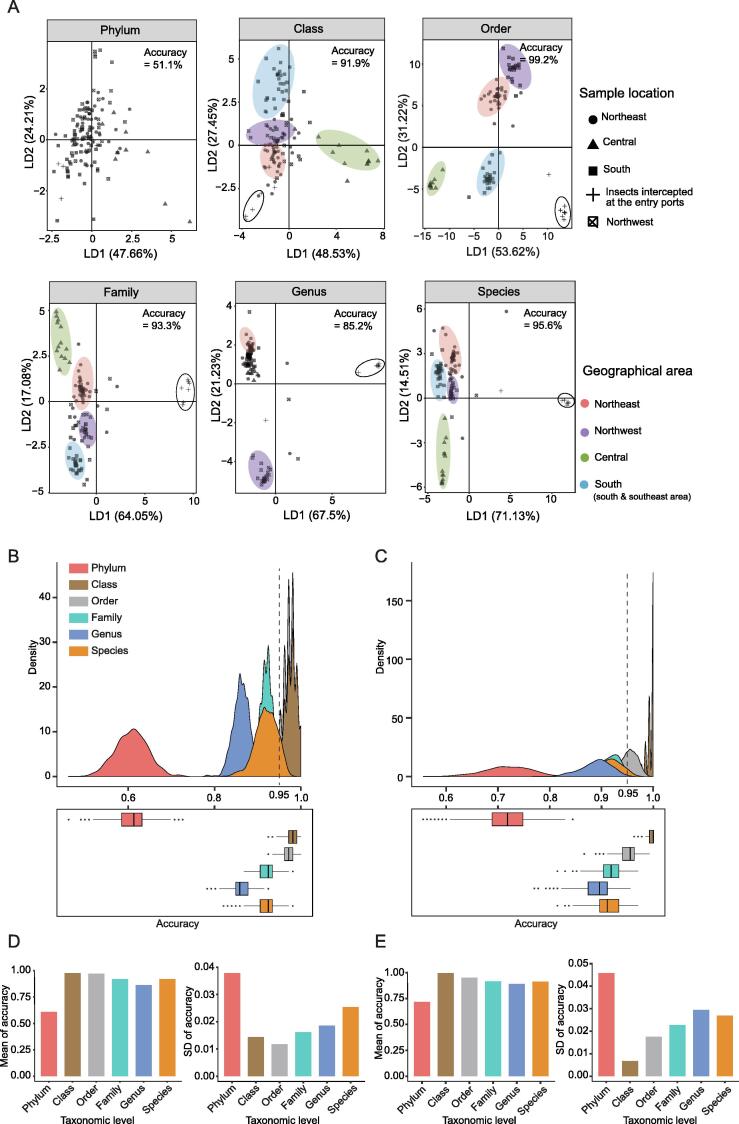
**Ability of LDA to distinguish the geographical clusters of all insects at each microbial taxonomic level** **A.** Performance of LDA method for distinguishing the geographical clusters of all insects at each microbial taxonomic level. Insect samples were shaped and circled according to their geographical areas. The transparent circle delineates several insects that were intercepted at entry ports. **B.** Accuracy distribution at each taxonomic level of gut microbes via the jackknife method. **C.** Accuracy distribution at each taxonomic level of gut microbes via bootstrapping. **D.** and **E.** Mean and SD of accuracy for jackknife (D) and bootstrap (E) methods. SD, standard deviation.

To ascertain the ability of LDA to determine the taxonomic level of microbes based on geographical information, we performed LDA at all taxonomic levels of microbes except for the kingdom level. The insects from the same geographical area were grouped at the class, order, family, and species levels; however, the insects from different geographical areas could not be distinguished at the phylum and genus levels (Figure 4A). The accuracies at the phylum, class, order, family, genus, and species levels were 51.1%, 91.9%, 99.2%, 93.3%, 85.2%, and 95.6%, respectively. Moreover, the insects collected from the entry ports were located far from those collected from other areas in China. To determine the robustness of this method, the jackknife method was performed 1000 times at all taxonomic levels, except for the kingdom level, by dropping approximately 15% of the sample each time. The accuracy distribution from the jackknife method showed that the accuracy at the phylum level was lowest, while those at the class and order levels were higher than 0.95 (Figure 4B). Bootstrapping was also performed 1000 times, and a similar result was obtained (Figure 4C). Class and order were considered proper taxonomic levels to distinguish the geographical clusters of host insects because of the high accuracy and low standard deviation in both jackknife and bootstrapping analyses (Figure 4D and E).

### LDA effectively distinguishes single quarantine insect species from different geographical sources at the phylum level

**Figure 5:**
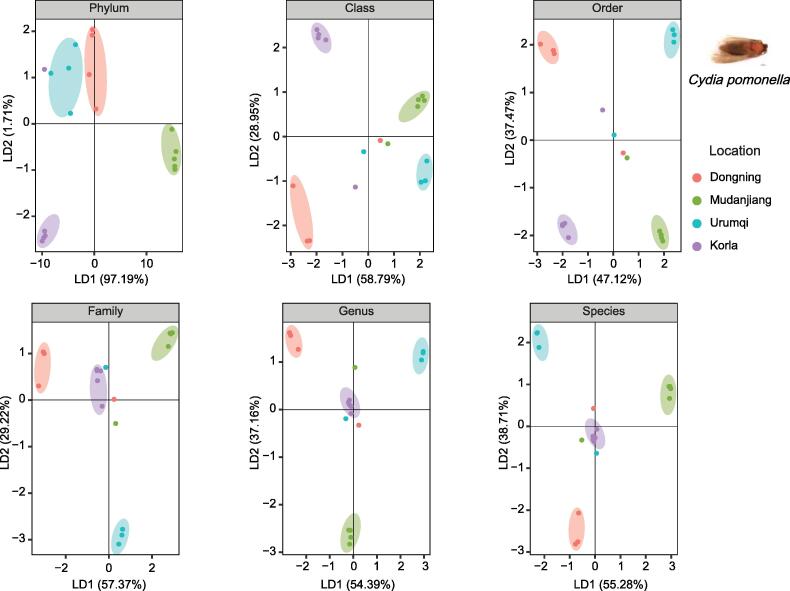
**Application of the LDA method for distinguishing *Cydia pomonella* samples of different geographical sources at each taxonomic level of gut microbes** A total of 18 *Cydia pomonella* isolates were collected from four sites. Two sites, Dongning and Mudanjiang, are in the northeast of China, whereas Urumqi and Korla are in the northwest of China. Dots of the same color were grouped in clusters, showing that the four sampling sites could be distinguished at most taxonomic levels.

To study the relationship between the gut microbiota and geographical source of the host, the “insect species” factor was controlled, and LDA based on geographical information was performed for five insect species, including *C*. *pomonella*, *I*. *typographus*, *L*. *decemlineata*, *L*. *oryzophilus*, and *Henosepilachna vigintioctopunctata*. Samples for each species were obtained from at least three areas. The LDA results for *C. pomonella* were similar to those for the other four selected insect species (Figure 5, Figures S4 and S5). For *C. pomonella*, 18 samples were collected from four sites. Two sites, Dongning and Mudanjiang, are located in the northeast of China, while Urumqi and Korla are located in the northwest of China. For all five species, the overall discriminating ability was significant at the above-genus level, with the phylum level exhibiting the best performance (phylum level, *P* = 0.009; class level, *P* = 0.027; order level, *P* = 0.02; family level, *P* = 0.022; Figure S6A and G). However, for a single species, the discriminating ability was significant at the above-family level (Figure S6B–F). For example, the geographical distribution of *H. vigintioctopunctata* could not be discriminated at the family level of the gut microbiota (*P* = 0.217; Figures S5B and S6F); the geographical distributions of *I. typographus* (*P* = 0.143; Figures S4A and S6C), *L. oryzophilus* (*P* = 0.177; Figures S5A and S6E), and *H. vigintioctopunctata* (*P* = 0.252; Figures S5B and S6F) could not be discriminated at the genus level; and the geographical distributions of *L. oryzophilus* (*P* = 0.229; Figures S5A and S6E) and *H. vigintioctopunctata* (*P* = 0.157; Figures S5B and S6F) could not be discriminated at the species level. Taken together, the phylum level is the best for tracing the geographical sources of a single insect species (Figure S6G).

**Figure 6:**
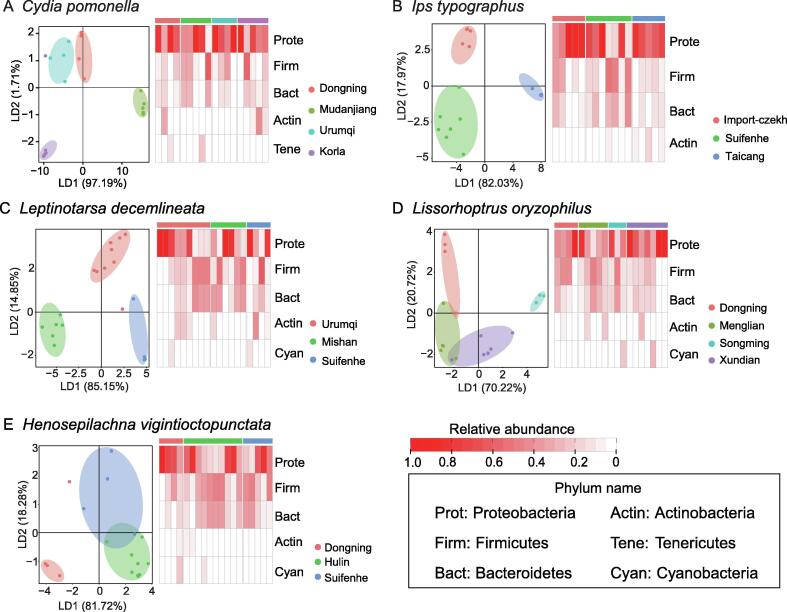
**LDA and heatmap for gut microbes of five representative insect species** The heatmap showed the relative abundance of gut microbes at the phylum level for each insect species. The bar at the top of the heatmap represents the geographical sources of the samples for each insect species. The geographical sources of the samples for each of the five insect species were analyzed at the phylum level of the gut microbiota. The geographical sources of *Cydia pomonella* (**A**), *Ips typographus* (**B**), and *Leptinotarsa decemlineata* (**C**) were traced accurately. For *Lissorhoptrus oryzophilus* (**D**), there was an overlap between samples collected from Xundian and Menglian, and both sites are located in Yunnan Province. For *Henosepilachna vigintioctopunctata* (**E**), samples collected from Suifenhe were overlapped with those from Hulin, although Suifenhe is geographically closer to Dongning than to Hulin.

Next, the geographical sources of the samples from each insect species mentioned above were analyzed at the phylum level of the gut microbiota. The geographical sources of the samples from *C. pomonella* (*R*^2^ = 0.88, *P* = 0.01; Figure 6A, Figure S6H), *I. typographus* (*R*^2^ = 0.86, *P* = 0.004; Figure 6B, Figure S6I), and *L. decemlineata* (*R*^2^ = 0.86, *P* = 0.002; Figure 6C, Figure S6J) were traced accurately. For *L. oryzophilus*, there was an overlap between the samples from Xundian and Menglian (*R*^2^ = 0.59, *P* = 0.002; Figure 6D, Figure S6K); both sampling sites are located in Yunnan Province. For *H. vigintioctopunctata*, the samples from Suifenhe were overlapped with those from Hulin (*R*^2^ = 0.63, *P* = 0.001; Figure 6E, Figure S6L), although Suifenhe is closer to Dongning than to Hulin in geography. A heatmap was used to find the most relevant microbial phylum to the geographical source of the host. One or several microbial phyla maybe not enough to prove geographical correlation, but in this study, there is evidence that the host geographical source is different at the phylum level (Figure 6).

## Discussion

To the best of our knowledge, this is the first attempt to trace the origins of quarantine insects based on their gut microbiota. There is currently no effective and rapid method available to identify the geographical sources of quarantine insects. Although the methods of DNA barcoding and RFLPs can predict geographical origins, numerous insect generations are required to detect gene flow from multiple origins [15], [16]. These methods are adequate for identifying the genetics of evolutionary relationships or for studying species complexes [17], [18].

Several methods have been evaluated in this study. PCA is not the most appropriate approach for extracting geographical information because the species, sex, developmental stage, diet, and geographical source of the host affect the gut microbiota, whereas the geographical source of the insect may not be the principal factor. Methods based on prior information, such as LDA, are better for distinguishing insects based on their geographical sources using gut microbiota. Geographical factors were revealed by the composition patterns of gut microbes. Supervised machine learning is effective in extracting composition patterns from data using prior information to establish a prediction model. Therefore, supervised machine learning was recommended. Some outliers were observed in the LDA because of prior information. The sampling sites of insects were defined as geographical sources. However, insects might have originated in other places, and their gut microbiota may not have been localized. This situation is inevitable in sampling; however, an increased number of samples and a robust algorithm will help reduce this noise.

We found that different insect species could be effectively distinguished at the phylum level of gut microbiota. Single insect species from different regions could be effectively distinguished at the phylum level. For invasive species in China with limited distributions, such as *C. pomonella*, this method can easily and quickly identify the source of the invasion. Similarly, for widely distributed species, such as *I. typographus*, this method can distinguish specimens from China and abroad. This is a problem that cannot be addressed using genetic tests or morphology. Moreover, insect gut microbiota is closely related to host sex, diet, developmental stage, and niche occupation. For example, honeybees living in different locations can differ considerably in their gut microbiota composition. The structure of the microbial community can also differ among bees depending on whether they forage on flowering rape crops [19]. Worker honeybees and solitary bees also have different gut microbiota. There are eight distinct bacterial species or phylotypes in worker honeybees: three are gram-positive species, such as *Bifidobacterium*, and five are gram-negative species, including β-proteobacterium [20]. Among predatory insects, the diversity of the prey consumed can increase the diversity of bacteria in the gut [21]. The diversity of microorganisms is also greatly influenced by differences in the plant species consumed [22], [23], [24]. The gut community of *L. decemlineata* larvae feeding on tomatoes was dominated by the genera *Stenotrophomonas* and *Lactococcus*; however, that of larvae feeding on potatoes was dominated by *Enterobacter* [25].

The accuracy of 16S rRNA gene sequencing is affected by the sequencing depth and coverage. The sequencing results showed that differences in gut microbiota at the phylum level could distinguish insect species, and differences at the phylum, class, and order levels could distinguish the same insect species from different source locations. It is feasible to trace the origin of insects from different geographical sources using the phylum/class/order-level gut microbes. However, owing to the limited number of sequencing samples, the LDA values of some of the samples did not cluster. These included *C. pomonella* collected from Ili and *L. decemlineata* collected from Urumqi. However, for *C. pomonella* and *I. typographus*, native individuals and intercepted conspecifics were distinguishable in this study.

We also addressed the problem of determining the gut microbiota composition of small insects. The body length of different insect species varies considerably. Most studies on insect gut microbes have dissected the gut and evaluated the interaction between the microbiota and host stage for bacterial identification [26], [27], [28], [29], [30]. However, it is difficult to obtain individual guts from small insects. Some individuals represented one sample from which genomic DNA was extracted, and the microbiota was amplified from the entire insect body. This approach helps solve the problem of individual differences and small sample sizes. The method of genomic DNA extraction from insect gut microbiota requires further study to establish better standards for future research.

In summary, we developed a method to trace the geographical origins of quarantine insects using prior information-based LDA. Bacteroidetes, Firmicutes, and Proteobacteria were the three dominant phyla in insect guts, and their relative abundances differed among insect species. The class and order levels of the gut microbiota can provide geographical information, even though gut microbiota is masked by insect species differences. The phylum, class, and order levels of gut microbiota are useful taxonomic levels for single insect species. The main purpose of this study was to provide ideas for new geographical traceability methods; however, the application of these methods still requires several key steps. First, real-time intelligent monitoring equipment should be developed using advanced information technologies such as the Internet of Things, which helps us find the invader in time. Second, gut microbial databases of different pests in different regions should be established as a reference. Finally, an integrated intelligent platform combined with the LDA algorithm and intelligent judgment should be developed to realize rapid and accurate traceability. Among these, the most difficult is to set up gut microbial databases. The gut microbiota in insects is complex and variable. The quantity and quality of the basic database will greatly impact identification accuracy, especially for prior information-based methods. A high-quality reference database containing a large number of pests from different regions can improve the accuracy and stability of the identification model. To control batch effects and ensure database quality, sample-processing methods should be standardized.

## Materials and methods

### Sample preparation

Quarantine insects collected from China were used in the present study. The collection sites were determined based on previous monitoring records of quarantined insects. Most of the insects were captured using aerial nets. At each location, we collected at least 10 individual insects from each species. Each individual was alive before it was immersed into RNAlater Stabilization Solution (Catalog No. AM7021, Ambion, Austin, TX).

### DNA extraction and 16S rRNA gene sequencing

One large individual (>5 mm), five intermediate-sized individuals (2–5 mm), or 10 small individuals (<5 mm) were used as one sample for gut microbiota DNA extraction. Before extraction, the surface of each insect was sterilized with 70% ethanol and washed twice with sterilized phosphate buffer solution (PBS) [31]. The abdomens or whole insects were placed into a special EP tube weighing 0.3 g with 0.1 mm glass beads (Catalog No. 11079101, BioSpec, Oklahoma City, OK). DNA extraction was performed using the QIAamp Fast DNA Stool Mini Kit (Catalog No. 51604, Qiagen, Hilden, Germany). Samples were pre-treated before DNA extraction. Pre-treatment steps were as follows: 1.4 ml of InhibitEX buffer was first added into the EP tube, and the sample was ground in a bead beater for 1 min. The samples were then incubated at 95 °C for 10 min and subsequently re-ground for 2 min. Thereafter, samples were centrifuged for 1 min to obtain the pellet. This was followed by the steps described in the kit protocol. The concentration of the extracted DNA was measured using Nanodrop, and then the DNA was used as a template for PCR amplification.

The PCR reaction system used was the HiFi HotStart DNA Polymerase (Catalog No. KR0369, Kapa Biosystems, Boston, MA) based on a two-step PCR reaction. The first-step PCR amplification was performed using the primer pairs under the following conditions: a denaturing step at 95 °C for 5 min; followed by 20 cycles of 98 °C for 20 s, 52 °C for 30 s, and 72 °C for 30 s; and a final step of 5 min at 72 °C. The primer pairs were 5′-CCTACGGGNBGCASCAG-3′ (forward) and 5′-GACTACNVGGGTATCTAATCC-3′ (reverse). The PCR products were purified using the Agencourt AMPuer XP System Kit (Catalog No. A63880, Beckman Coulter, San Francisco, CA), and the purified products were used for the second-step PCR amplification under the conditions as follows: denaturing at 95 °C for 5 min; followed by 10 cycles of 98 °C for 20 s, 60 °C for 30 s, and 72 °C for 30 s; and a final step of 5 min at 72 °C. The primer pairs were Illumina sequencing joint with different indices, with V3 and V4 information of 16S RNA gene. PCR products were purified using the same protocol as described above. Thereafter, the concentration of each sample was detected after electrophoresis. Sequencing was performed using paired-end 250 bp (PE250) sequencing (HiSeq2500; Illumina, San Diego, CA).

### Quality control and taxonomy assignment

Following sequencing, sequences were distributed into samples based on barcodes. Following the removal of barcodes and primers, we trimmed 10 bp of sequences at the start and end of each read for quality control [32]. Sequences longer than 104 bp were retained after trimming bases whose quality was below 20 using Sickle (v1.33) software. Error correction was performed using SPAdes (v3.1.9) software [33].

The workflow “pick_open_reference_otus.py” in QIIME (v1.9.1) was used to select operational taxonomic units at 97% similarity and assign a species level using the UCLUST method in the Greengene database.

### Phylogenetic analysis

We downloaded the *COI* gene sequences of test insects from the Barcode of Life Data System database (https://www.barcodinglife.org) [34]. These 2374 sequences were aligned using Clustal Omega (https://www.ebi.ac.uk/Tools/msa/clustalo/). Then, a method based on maximum likelihood, RAxML-HPC2 (v8.2.10) [35], was used to construct a phylogenetic tree. We performed 1000 bootstrap replicates for this tree after removing suspicious sequences. The tree was edited and visualized using FigTree (v1.4.3).

### Data analysis and visualization

The density distribution of sequencing quantity and composition of gut microbiota was analyzed using R (v3.4.1). The alpha diversity index was calculated using the vegan package (v2.5-3) in R. PCA was performed using the R statistics package (v3.4.1). LDA was performed using the MASS package in R software. The heatmap was visualized using the R package pheatmap (v1.0.10).

PCA for insects collected from the five geographical areas was performed based on the relative abundance of gut microbes. LDA for all insects collected from the five geographical areas was performed based on the relative abundance normalized using the log function. LDA for reared insects and representative insects was performed using relative abundance. The first and second components were selected for visualization using the R package ggplot2. The ratio of the LDA classification results to the original sample information was defined as accuracy. The jackknife method was performed 1000 times, excluding ∼ 15% of the sample from each geographical source each time. Bootstrapping was performed 1000 times, and the number of bootstrap samples was equal to that of the original samples. A heatmap was constructed using relative abundance at the order level using the R package pheatmap. The distance between each group in LDA or PCA was measured using a permutational multivariate analysis of variance (PERMANOVA) in the R package vegan (v2.5-3). A comparison of ADONIS (*R*^2^) and *P* values between LDA and PCA was performed with *t*-test using the R package vegan (v2.5-3).

## Data availability

All sequencing data have been deposited in the Genome Sequence Archive [36] at the National Genomics Data Center, Beijing Institute of Genomics, Chinese Academy of Sciences / China National Center for Bioinformation (GSA: CRA002386), and are publicly accessible at https://ngdc.cncb.ac.cn/gsa.

## CRediT author statement

**Yanxue Yu:** Resources, Data curation, Writing - original draft. **Qi Wang:** Formal analysis, Writing - original draft. **Ping Zhou:** Investigation. **Na Lv:** Methodology. **Wei Li:** Software. **Fangqing Zhao:** Conceptualization, Methodology. **Shuifang Zhu:** Supervision. **Di Liu:** Conceptualization, Writing - review & editing. All authors have read and approved the final manuscript.

## Competing interests

The authors have declared no competing interests.

## Supplementary material

Supplementary Figure S1 **Diagnostic ability of PCA for tracing the geographical source of insects** Each point represents an insect, and the colored points represent the geographical area where the insect was collected. The hollow points represent imported insects.

Supplementary Figure S2 **LDA discriminating sex or species of insects based on gut microbes** LDA can distinguish between insects based on sex **(A)** and species differences **(B)**.

Supplementary Figure S3 **Discrimination and comparison of LDA and PCA using the PERMANOVA test** The methods of PCA (left panel) and LDA (right panel) were compared using the ADONIS test with reared insects whose geographical source and diet factors were controlled. **A.** Comparison of sex between the two methods. LDA was better able to discriminate between sexes compared to PCA. **B.** Comparison of the two methods for species factors. For discriminating insect species, *R*^2^ = 0.71, *P* = 0.001 for LDA; *R*^2^ = 0.10, *P* = 0.36 for PCA. These results suggest that LDA has better diagnostic ability than PCA for extracting specific factors affecting the gut microbiota.

Supplementary Figure S4 **Verification of ability of LDA to distinguish *I. typographus* and *L. decemlineata* at each microbial taxonomic level A.** LDA distinguished *I. typographus* at all taxonomic levels. The point represents an insect, the colored point shows its geographical source, and the same geographical area was clustered together. Red, green, and blue areas indicate samples from abroad, samples from Suifenhe in Heilongjiang Province, and samples from Taicang in Jiangsu Province, respectively **B.** LDA distinguished *L. decemlineata* at all taxonomic levels. The point represents an insect, and the colored point shows its geographical source, and the same geographical area was clustered together. Red, green, and blue areas indicate samples from Urumqi, Mishan, and Suifenhe in Heilongjiang Province, respectively.

Supplementary Figure S5 **Verification of ability of LDA to distinguish *L. oryzophilus* and *H. vigintioctopunctata* at each microbial taxonomic level A.** LDA distinguished *L. oryzophilus* at all taxonomic levels. The point represents an insect, the colored point shows its geographical source, and the same geographical area was clustered together. The red, green, blue, and purple areas indicate samples from Dongning in Heilongjiang Province, Menglian, Songming, and Xundian in Yunnan Province, respectively. **B.** LDA distinguished *H. vigintioctopunctata* at all taxonomic levels. The point represents an insect, the colored point shows its geographical source, and the same geographical area was clustered together. The red, green, and blue areas indicate samples from Dongning, Hulin, and Suifenhe in Heilongjiang Province, respectively.

Supplementary Figure S6 **PERMANOVA test for five insect species** PERMANOVA tests showed the relationship between each taxonomic level, samples sites pairwise (**A**), and median *P*-values at each taxonomic level (**G**). The median *P*-values for the five insect species *C. pomonella* (**B**), *I. typographus* (**C**), *L. decemlineata* (**D**), *L. oryzophilus* (**E**), and *H. vigintioctopunctata* (**F**). PERMANOVA test of the distance between each group in LDA for *C. pomonella* (**H**), *I. typographus* (**I**), *L. decemlineata* (**J**), *L. oryzophilus* (**K**), and *H. vigintioctopuntata* (**L**).
