# Supplementary figures and images for: First Glimpse of Gut Microbiota of Quarantine Insects in China
Source: Genomics Proteomics Bioinformatics. 2022 May 24;20(2):394–404. doi: 10.1016/j.gpb.2022.04.005 (PMC9684152; doi:10.1016/j.gpb.2022.04.005)

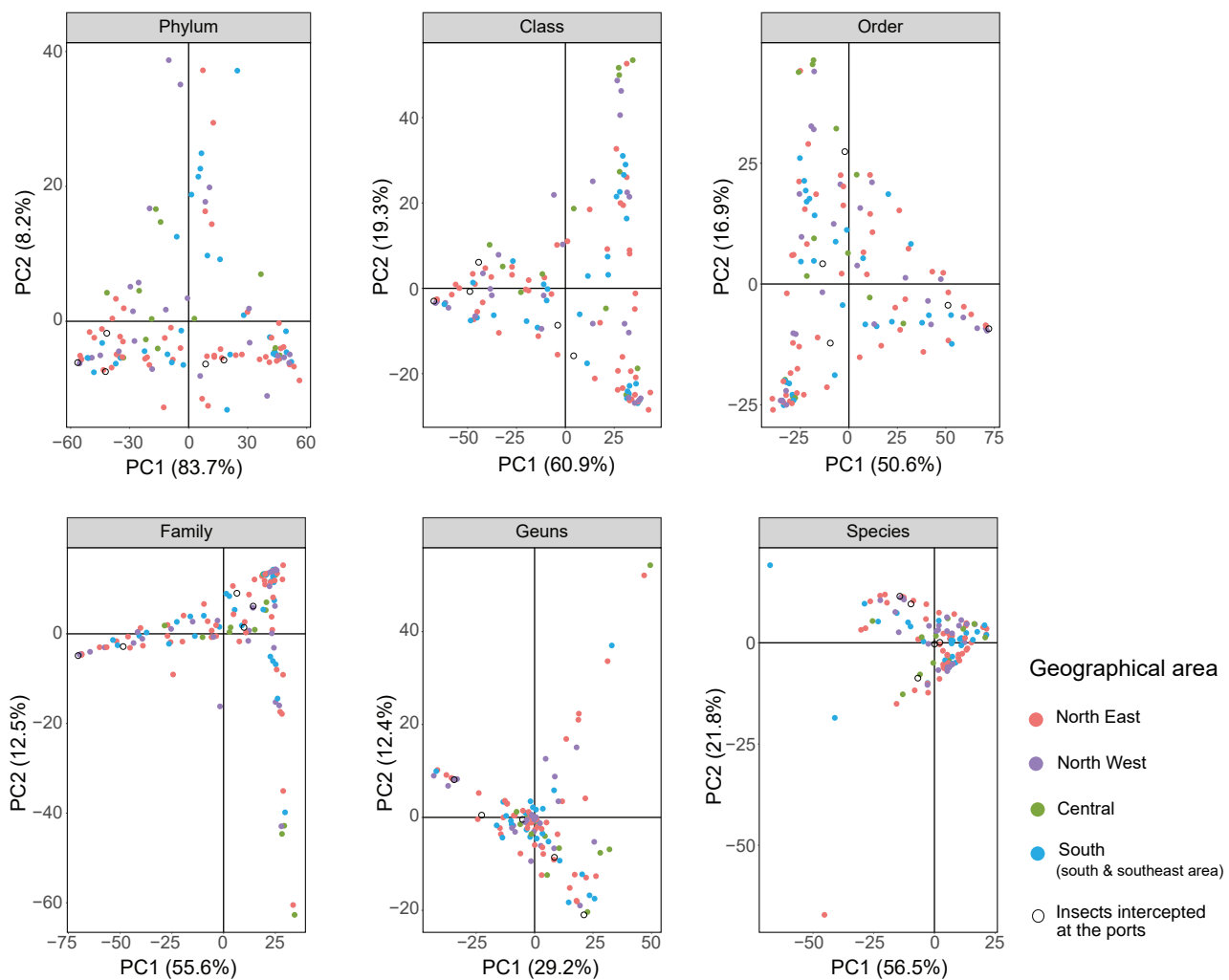

Supplement: Supplementary Figure S1 — Diagnostic ability of PCA for tracing the geographical source of insects Each point represents an insect, and the colored points represent the geographical area where the insect was collected. The hollow points represent imported insects. [file mmc1.pdf]

A

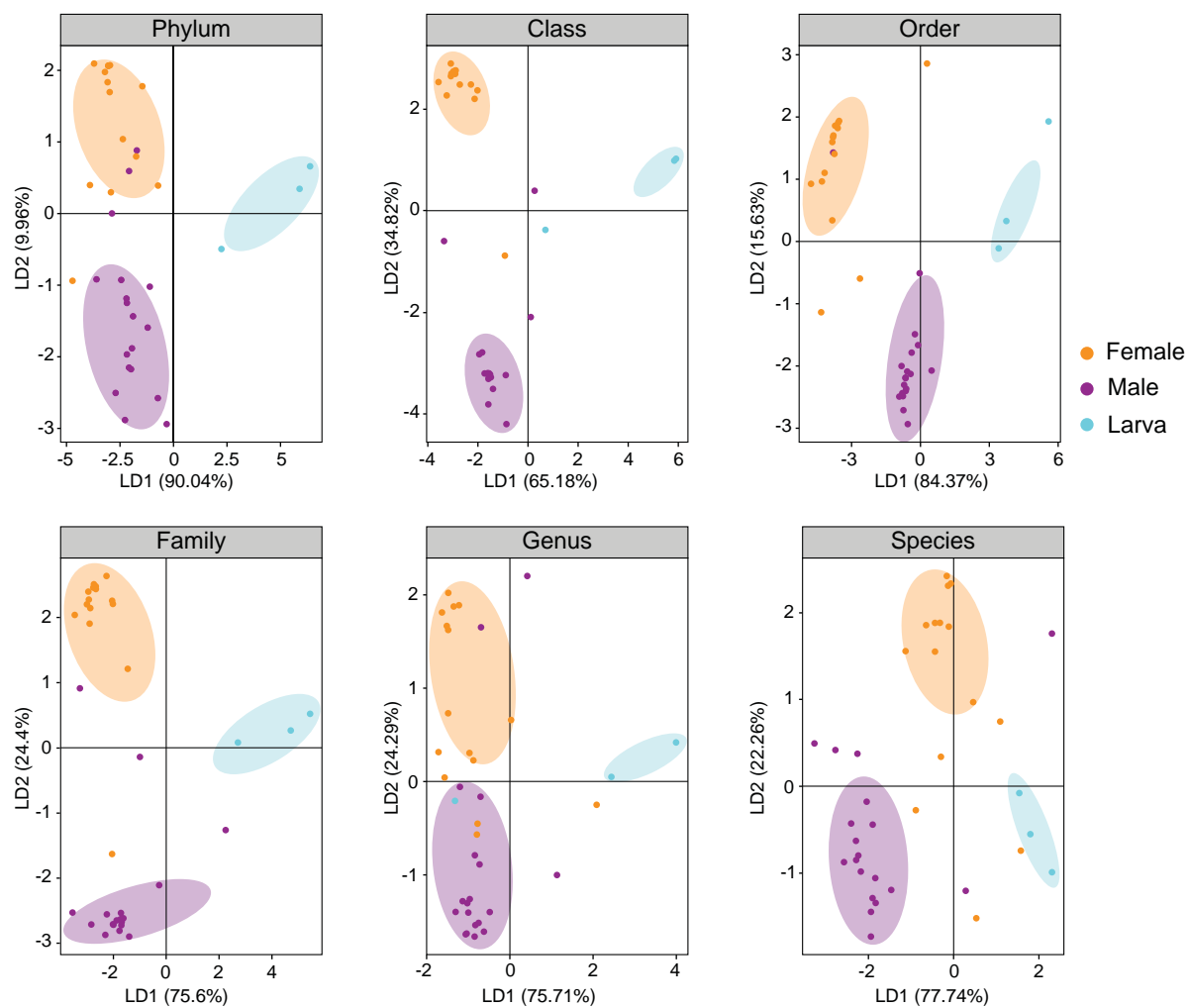

B

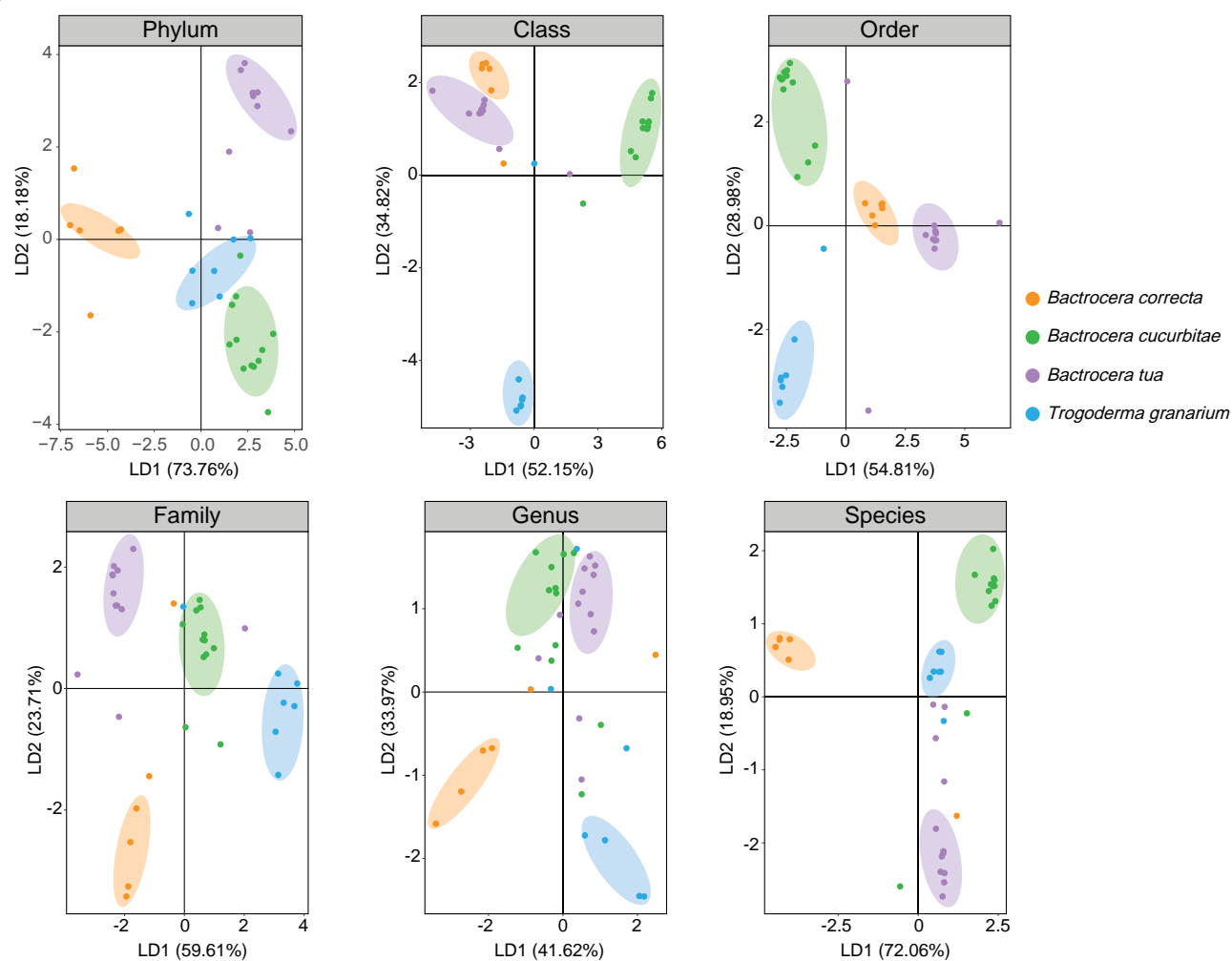

Supplement: Supplementary Figure S2 — LDA discriminating sex or species of insects based on gut microbes LDA can distinguish between insects based on sex (A) and species differences (B). [file mmc2.pdf]

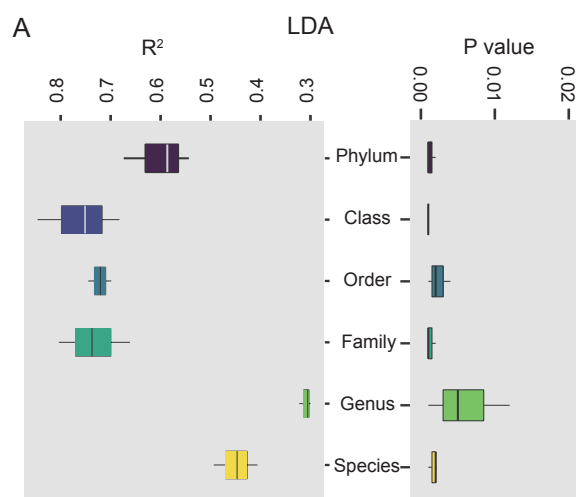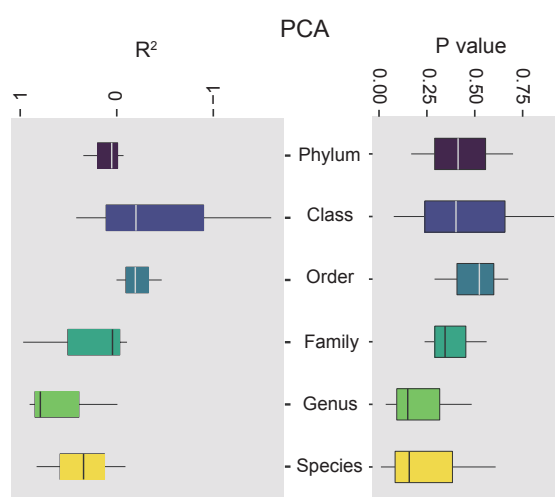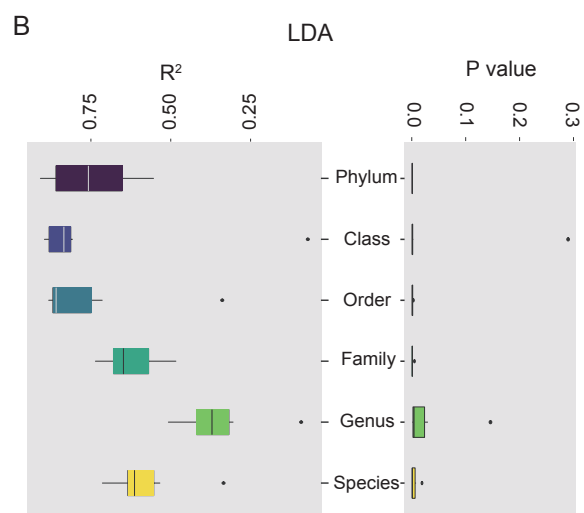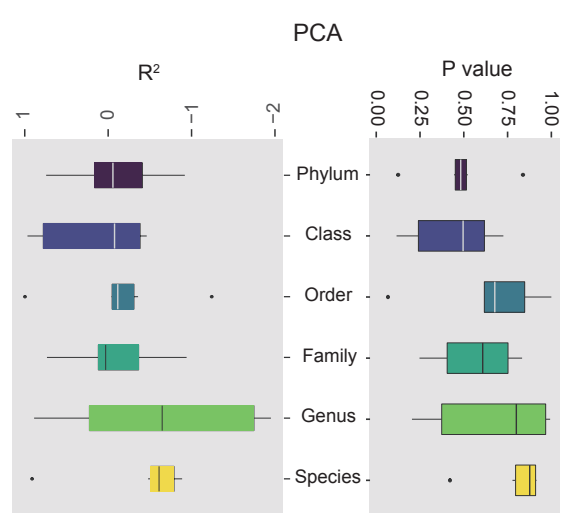

Supplement: Supplementary Figure S3 — Discrimination and comparison of LDA and PCA using the PERMANOVA test The methods of PCA (left panel) and LDA (right panel) were compared using the ADONIS test with reared insects whose geographical source and diet factors were controlled. A. Comparison of sex between the two methods. LDA was better able to discriminate between sexes compared to PCA. B. Comparison of the two methods for species factors. For discriminating insect species, R2 = 0.71, P = 0.001 for LDA; R2 = 0.10, P = 0.36 for PCA. These results suggest that LDA has better diagnostic ability than PCA for extracting specific factors affecting the gut microbiota. [file mmc3.pdf]

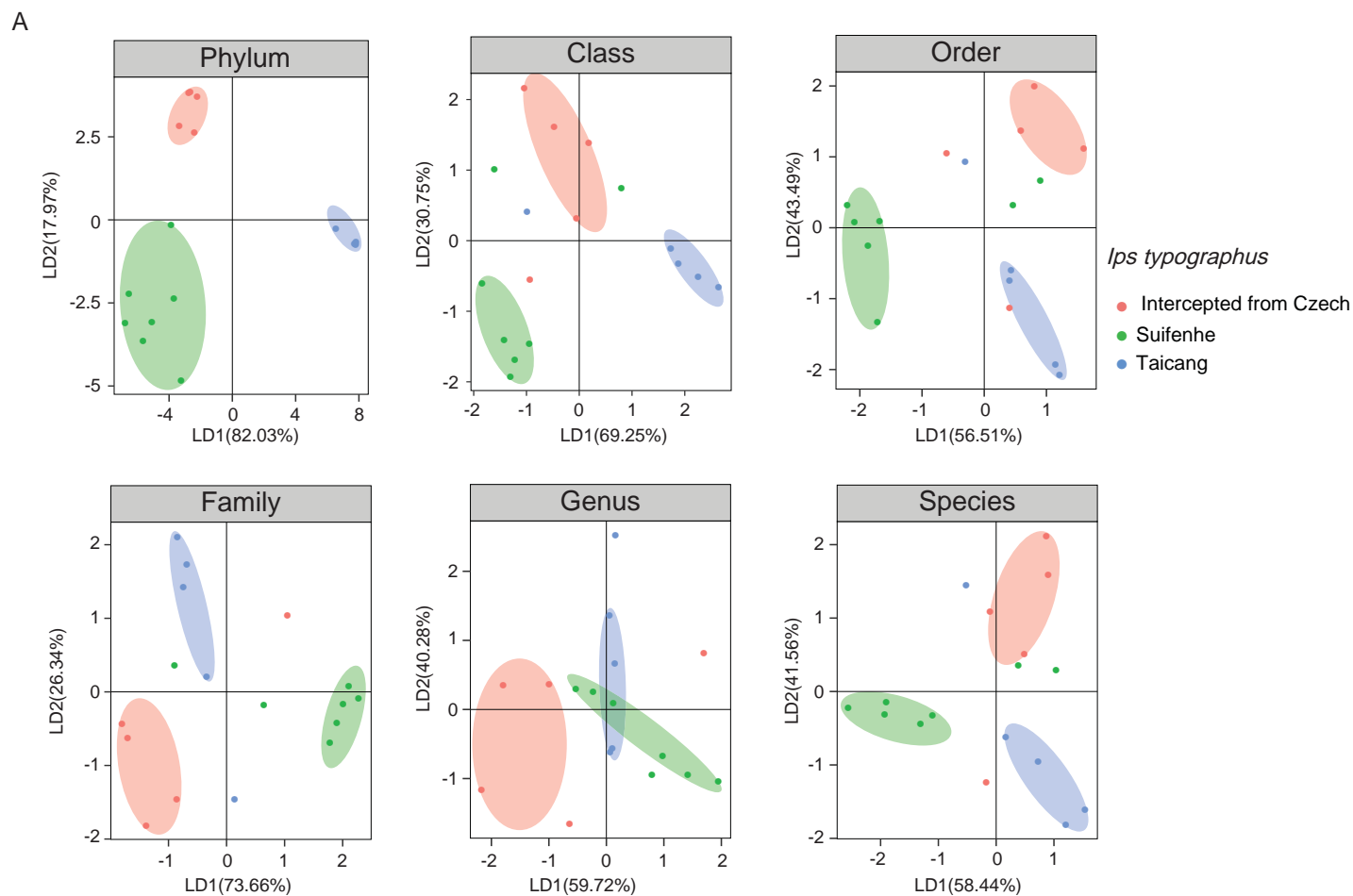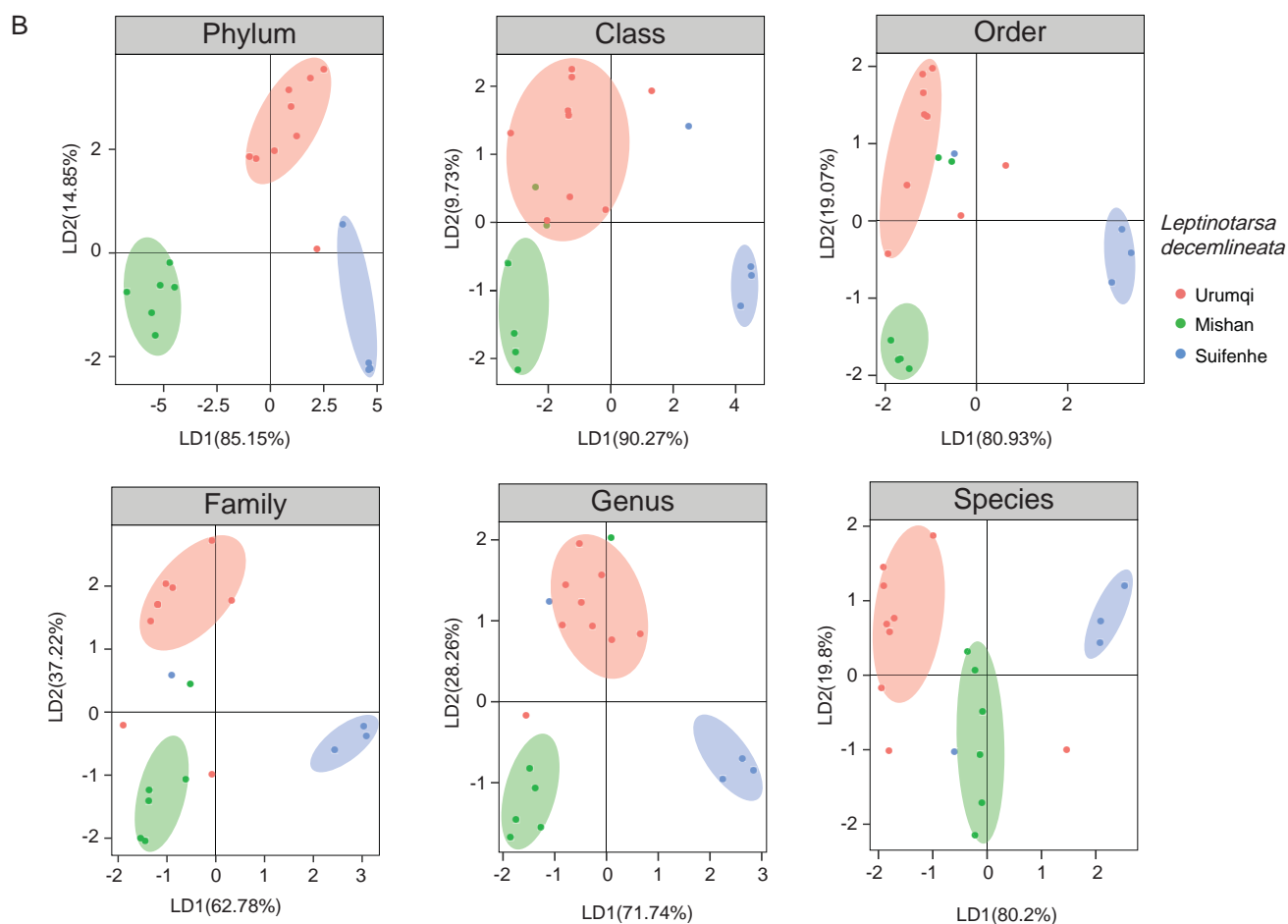

Supplement: Supplementary Figure S4 — Verification of ability of LDA to distinguish I. typographus and L. decemlineata at each microbial taxonomic level A. LDA distinguished I. typographus at all taxonomic levels. The point represents an insect, the colored point shows its geographical source, and the same geographical area was clustered together. Red, green, and blue areas indicate samples from abroad, samples from Suifenhe in Heilongjiang Province, and samples from Taicang in Jiangsu Province, respectively B. LDA distinguished L. decemlineata at all taxonomic levels. The point represents an insect, and the colored point shows its geographical source, and the same geographical area was clustered together. Red, green, and blue areas indicate samples from Urumqi, Mishan, and Suifenhe in Heilongjiang Province, respectively. [file mmc4.pdf]

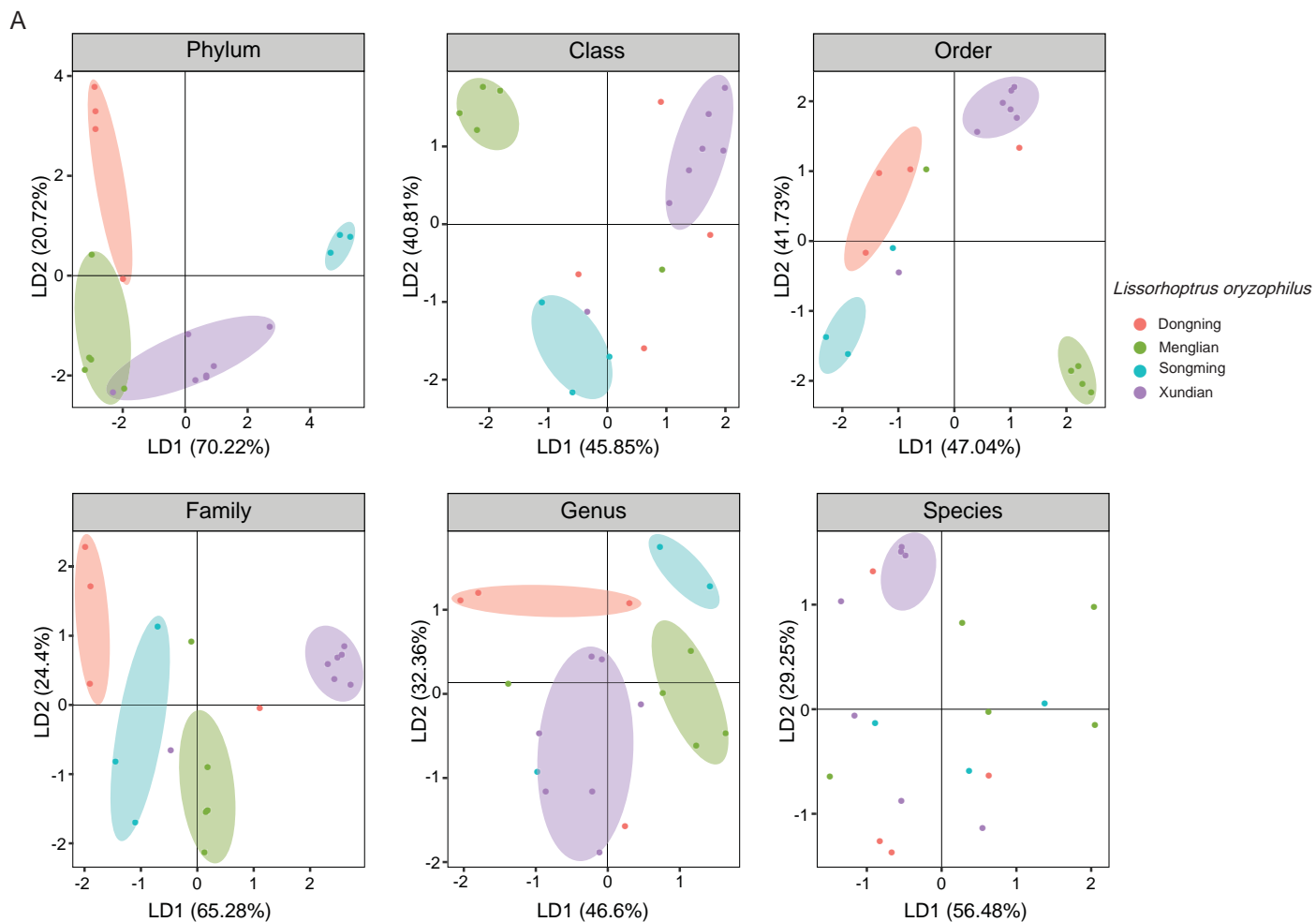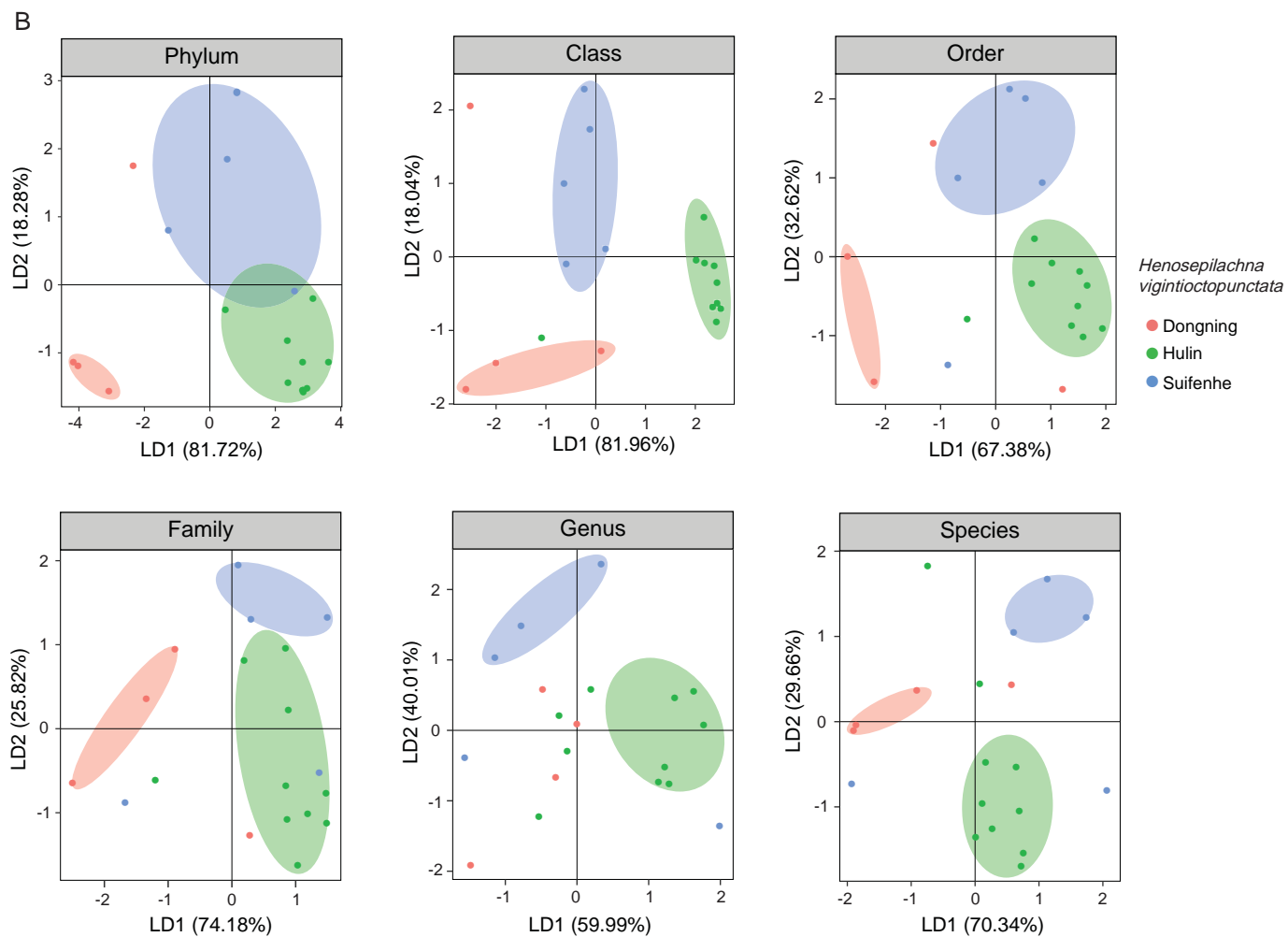

Supplement: Supplementary Figure S5 — Verification of ability of LDA to distinguish L. oryzophilus and H. vigintioctopunctata at each microbial taxonomic level A. LDA distinguished L. oryzophilus at all taxonomic levels. The point represents an insect, the colored point shows its geographical source, and the same geographical area was clustered together. The red, green, blue, and purple areas indicate samples from Dongning in Heilongjiang Province, Menglian, Songming, and Xundian in Yunnan Province, respectively. B. LDA distinguished H. vigintioctopunctata at all taxonomic levels. The point represents an insect, the colored point shows its geographical source, and the same geographical area was clustered together. The red, green, and blue areas indicate samples from Dongning, Hulin, and Suifenhe in Heilongjiang Province, respectively. [file mmc5.pdf]

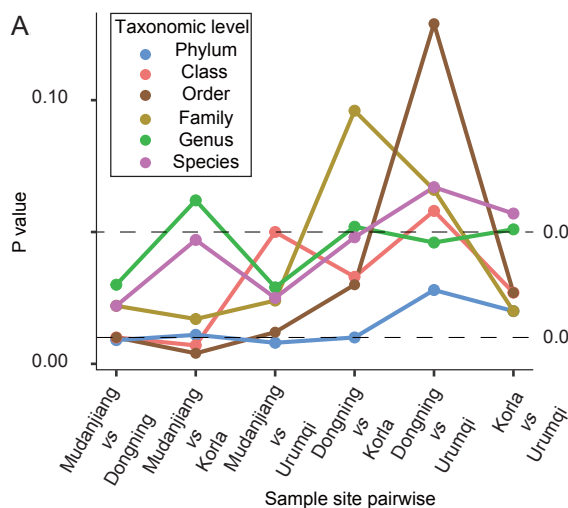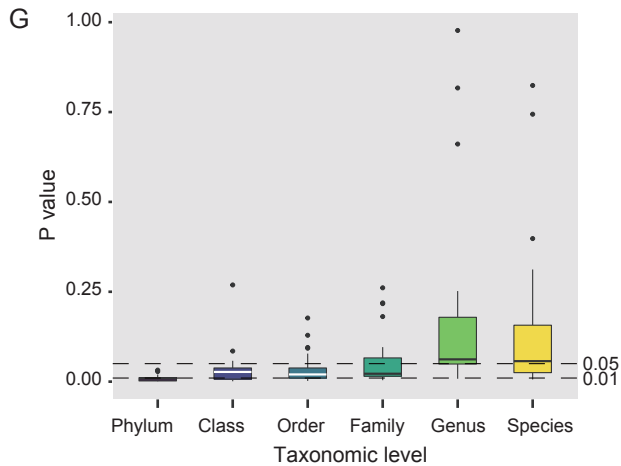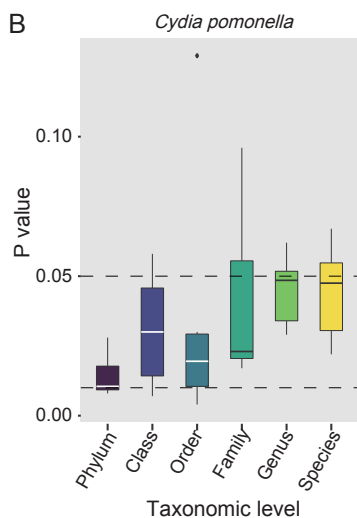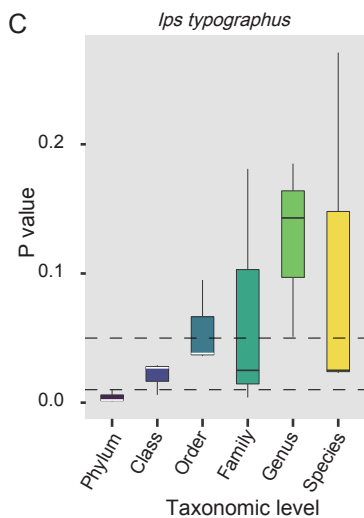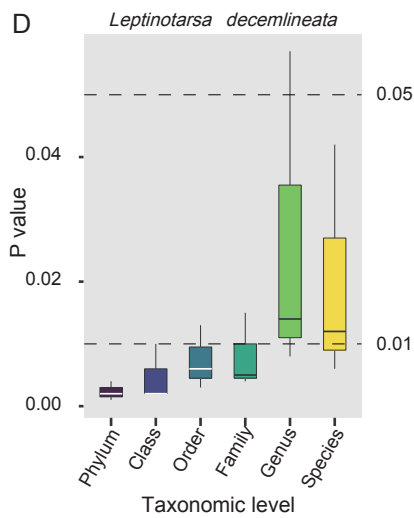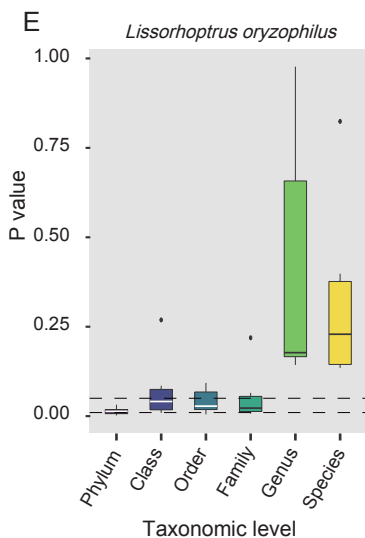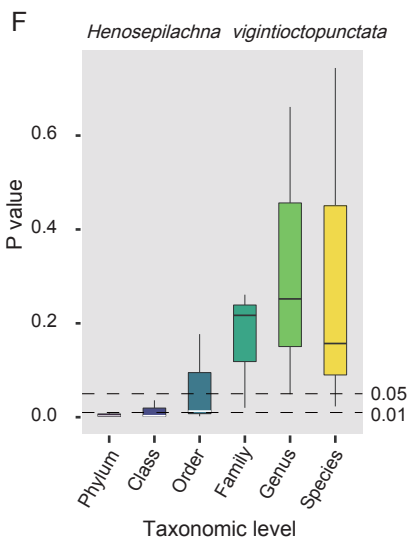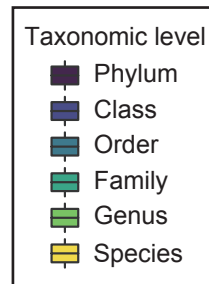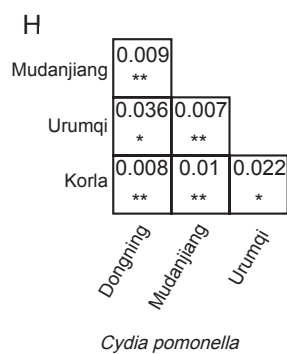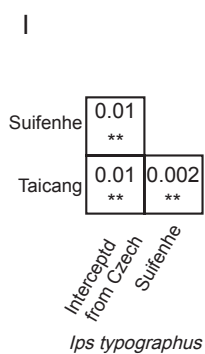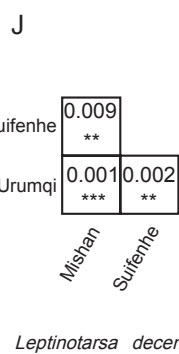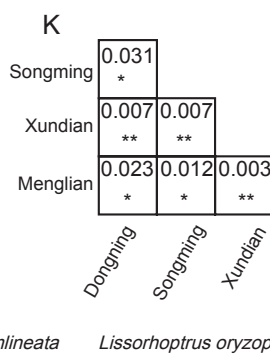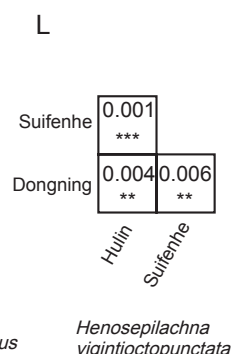

Supplement: Supplementary Figure S6 — PERMANOVA test for five insect species PERMANOVA tests showed the relationship between each taxonomic level, samples sites pairwise (A), and median P-values at each taxonomic level (G). The median P-values for the five insect species C. pomonella (B), I. typographus (C), L. decemlineata (D), L. oryzophilus (E), and H. vigintioctopunctata (F). PERMANOVA test of the distance between each group in LDA for C. pomonella (H), I. typographus (I), L. decemlineata (J), L. oryzophilus (K), and H. vigintioctopuntata (L). [file mmc6.pdf]
