# Supplementary material for: First Glimpse of Gut Microbiota of Quarantine Insects in China
Source: Genomics Proteomics Bioinformatics. 2022 May 24;20(2):394–404. doi: 10.1016/j.gpb.2022.04.005 (PMC9684152; doi:10.1016/j.gpb.2022.04.005)
Supplement: Supplementary Table S1 [file mmc7.docx]

**Table S1 Detailed information on insect samples from different locations in China**

| Sample number | Scientific name | Collection site | Habitat | Diet | Sex | Development stage |
| --- | --- | --- | --- | --- | --- | --- |
| 1 | *Planococcus minor* | Guangzhou, Guangdong, China | Guava tree | *Psidium guajava* | Male/Female | Adult |
| 2 | *Lissorhoptrus oryzophilus* | Dongning, Heilongjiang, China | Paddy | *Oryza sativa* | Male/Female | Adult |
| 2 | *Lissorhoptrus oryzophilus* | Menglian, Yunnan, China | Paddy | *Oryza sativa* | Male/Female | Adult |
| 2 | *Lissorhoptrus oryzophilus* | Songming, Yunnan, China | Paddy | *Oryza sativa* | Male/Female | Adult |
| 2 | *Lissorhoptrus oryzophilus* | Xundian, Yunnan, China | Paddy | *Oryza sativa* | Male/Female | Adult |
| 3 | *Bactrocera correcta* | Beijing, China (Lab rearing) | Lab | Artificial feed | Male/Female | Adult |
| 4 | *Phenacoccus solenopsis* | Guangzhou, Guangdong, China | Cotton field | *Gossypium spp.* | Male/Female | Adult |
| 5 | *Trogoderma granarium* | Beijing, China (Lab rearing) | Lab | Artificial feed | Male/Female | Adult/Larva |
| 6 | *Bactrocera cucurbitae* | Beijing, China (Lab rearing) | Lab | Artificial feed | Male/Female | Adult |
| 7 | *Solenopsis invicta* | Jinghong, Yunnan, China | Park | Omnivorous | Male/Female | Adult/Larva |
| 8 | *Dendroctonus pseudotsugae* | Taicang, Jiangsu, China | Coniferous Tree | *Pseudotsuga sinensis* | Male/Female | Adult |
| 9 | *Bactrocera dorsalis* | Beijing, China (Lab rearing) | Lab | Artificial feed | Male/Female | Adult |
| 10 | *Leptinotarsa decemlineata* | Urumqi, the Xinjiang Uygur Autonomous Region, China | Potato field | *Solanum tuberosum* | Male/Female | Adult |
| 10 | *Leptinotarsa decemlineata* | Suifenhe, Heilongjiang, China | Potato field | *Solanum tuberosum* | Male/Female | Adult |
| 10 | *Leptinotarsa decemlineata* | Mishan, Heilongjiang, China | Potato field | *Solanum tuberosum* | Male/Female | Adult |
| 11 | *Henosepilachna vigintioctopunctata* | Dongning, Heilongjiang, China | Potato field | *Solanum tuberosum* | Male/Female | Adult |
| 11 | *Henosepilachna vigintioctopunctata* | Hulin, Heilongjiang, China | Potato field | *Solanum tuberosum* | Male/Female | Adult/Larva |
| 11 | *Henosepilachna vigintioctopunctata* | Suifenhe, Heilongjiang, China | Potato field | *Solanum tuberosum* | Male/Female | Adult |
| 12 | *Bactrocera tau* | Beijing, China (Lab rearing) | Lab | Artificial feed | Male/Female | Adult |
| 13 | *Platypus parallelus* | Yangzhou, Jiangsu, China (Intercepted from the Solomon islands) | Wooden packing | Broad -leaved wood | Male/Female | Adult |
| 14 | *Cydia pomonella* | Dongning, Heilongjiang, China | Apple tree | *Malus pumila* | Male/Female | Adult |
| 14 | *Cydia pomonella* | Mudanjiang, Heilongjiang, China | Apple tree | *Malus pumila* | Male/Female | Adult |
| 14 | *Cydia pomonella* | Urumqi, the Xinjiang Uygur Autonomous Region, China | Apple tree | *Malus pumila* | Male/Female | Adult |
| 14 | *Cydia pomonella* | Korla, the Xinjiang Uygur Autonomous Region, China | Apple tree | *Malus pumila* | Male/Female | Adult |
| 14 | *Cydia pomonella* | Ili, the Xinjiang Uygur Autonomous Region, China | Apple tree | *Malus pumila* | Male/Female | Adult |
| 14 | *Cydia pomonella* | Urumqi, the Xinjiang Uygur Autonomous Region, China (Intercepted from Kazakhstan) | Apple tree | *Malus pumila* | Male/Female | Larva |
| 15 | *Eriosoma lanigerum* | Menglian, Yunnan, China | Apple tree | *Malus pumila* | Male/Female | Adult/Larva |
| 16 | *Lymantria dispar* | Beijing, China (Lab rearing) | Lab | Artificial feed | Male/Female | Adult |
| 17 | *Dysmicoccus neobrevipes* | Guangzhou, Guangdong, China | Banana orchard | *Musa nana* | Male/Female | Adult |
| 18 | *Brontispa longissima* | Haikou, Hainan, China | Coconut Tree | *Cocos nucifera* | Male | Adult |
| 19 | *Opisina arenosella* | Haikou, Hainan, China | Coconut Tree | *Cocos nucifera* | Male/Female | Adult |
| 20 | *Sitophilus zeamais* | Shanghai, China | Corn seed | *Zea mays* | Male/Female | Adult |
| 21 | *Ips typographus* | Taicang, Jiangsu, China | Spruce | *Picea koraiensis* | Male/Female | Adult |
| 21 | *Ips typographus* | Huangdao, Shandong, China (Intercepted from Czech) | Spruce | *Picea koraiensis* | Male/Female | Adult |
| 21 | *Ips typographus* | Suifenhe, Heilongjiang, China | Spruce | *Picea koraiensis* | Male/Female | Adult |
| 22 | *Carpomya vesuviana* | Ili, the Xinjiang Uygur Autonomous Region, China | Jujube tree | *Ziziphus jujuba* | Male/Female | Adult |
