# Supplementary material for: First Glimpse of Gut Microbiota of Quarantine Insects in China
Source: Genomics Proteomics Bioinformatics. 2022 May 24;20(2):394–404. doi: 10.1016/j.gpb.2022.04.005 (PMC9684152; doi:10.1016/j.gpb.2022.04.005)
Supplement: Supplementary Table S2 [file mmc8.docx]

**Table S2 Detailed information of 179 gut microbiota samples for 16S rDNA sequencing**

| Sample ID | Scientific name | Collection sites | DNA concentration (ng/μL) | Raw data (Mbp) | Clean data (Mbp) | Raw reads | Clean reads |
| --- | --- | --- | --- | --- | --- | --- | --- |
| 205 | *Planococcus minor* | Guangzhou, Guangdong, China | 23.68 | 22 | 19 | 69,454 | 59,587 |
| 206 | *Planococcus minor* | Guangzhou, Guangdong, China | 64.23 | 32 | 28 | 102,550 | 89,754 |
| 36 | *Lissorhoptrus oryzophilus* | Dongning, Heilongjiang, China | 22.22 | 26 | 24 | 87,298 | 76,426 |
| 37 | *Lissorhoptrus oryzophilus* | Dongning, Heilongjiang, China | 22.13 | 30 | 25 | 94,464 | 81,592 |
| 38 | *Lissorhoptrus oryzophilus* | Dongning, Heilongjiang, China | 14.68 | 24 | 21 | 78,560 | 69,385 |
| 39 | *Lissorhoptrus oryzophilus* | Dongning, Heilongjiang, China | 11.01 | 18.6 | 17 | 61,918 | 51,086 |
| 122 | *Lissorhoptrus oryzophilus* | Menglian, Yunnan, China | 5.84 | 26 | 22 | 80,860 | 67,087 |
| 123 | *Lissorhoptrus oryzophilus* | Menglian, Yunnan, China | 5.96 | 30 | 26 | 99,824 | 77,654 |
| 190 | *Lissorhoptrus oryzophilus* | Menglian, Yunnan, China | 3.87 | 28 | 25 | 91,726 | 79,422 |
| 191 | *Lissorhoptrus oryzophilus* | Menglian,Yunnan, China | 2.84 | 26 | 22 | 81,492 | 70,433 |
| 192 | *Lissorhoptrus oryzophilus* | Menglian, Yunnan, China | 8.63 | 26 | 24 | 86,096 | 73,955 |
| 193 | *Lissorhoptrus oryzophilus* | Songming, Yunnan, China | 4.73 | 30 | 26 | 94,114 | 81,426 |
| 194 | *Lissorhoptrus oryzophilus* | Songming, Yunnan, China | 4.8 | 24 | 21 | 77,630 | 67,053 |
| 195 | *Lissorhoptrus oryzophilus* | Songming, Yunnan, China | 5.18 | 28 | 25 | 91,036 | 79,774 |
| 53 | *Lissorhoptrus oryzophilus* | Xundian, Yunnan, China | 17.11 | 36 | 31 | 116,608 | 98,949 |
| 54 | *Lissorhoptrus oryzophilus* | Xundian, Yunnan, China | 12.06 | 38 | 33 | 124,876 | 102,255 |
| 55 | *Lissorhoptrus oryzophilus* | Xundian, Yunnan, China | 5.35 | 24 | 20 | 77,240 | 60,928 |
| 153 | *Lissorhoptrus oryzophilus* | Xundian, Yunnan, China | 1.45 | 32 | 29 | 105,502 | 89,092 |
| 154 | *Lissorhoptrus oryzophilus* | Xundian, Yunnan, China | 1.13 | 26 | 24 | 86,758 | 74,504 |
| 155 | *Lissorhoptrus oryzophilus* | Xundian, Yunnan, China | 3.75 | 26 | 23 | 85,622 | 72,870 |
| 156 | *Lissorhoptrus oryzophilus* | Xundian, Yunnan, China | 1.56 | 18.6 | 17 | 62,034 | 54,202 |
| 161 | *Bactrocera correcta* | Beijing, China(Lab rearing) | 6.86 | 32 | 28 | 104,232 | 92,355 |
| 162 | *Bactrocera correcta* | Beijing, China(Lab rearing) | 7.14 | 36 | 32 | 117,478 | 99,571 |
| 163 | *Bactrocera correcta* | Beijing, China(Lab rearing) | 73.39 | 24 | 20 | 73,674 | 63,719 |
| 164 | *Bactrocera correcta* | Beijing, China(Lab rearing) | 9.35 | 19.8 | 18 | 66,182 | 57,700 |
| 199 | *Bactrocera correcta* | Beijing, China(Lab rearing) | 12.48 | 28 | 24 | 89,756 | 70,705 |
| 200 | *Bactrocera correcta* | Beijing, China(Lab rearing) | 12.4 | 24 | 22 | 80,138 | 70,080 |
| 207 | *Phenacoccus solenopsis* | Guangzhou, Guangdong, China | 25.36 | 26 | 22 | 82,318 | 71,654 |
| 208 | *Phenacoccus solenopsis* | Guangzhou, Guangdong, China | 37.4 | 28 | 24 | 88,752 | 77,993 |
| 10 | *Trogoderma granarium* | Beijing, China(Lab rearing) | 1.37 | 28 | 24 | 89,462 | 77,118 |
| 143 | *Trogoderma granarium* | Beijing, China(Lab rearing) | 1.83 | 28 | 25 | 92,210 | 80,209 |
| 144 | *Trogoderma granarium* | Beijing, China(Lab rearing) | 2.5 | 22 | 18 | 67,172 | 58,132 |
| 148 | *Trogoderma granarium* | Beijing, China(Lab rearing) | 6.79 | 32 | 29 | 106,696 | 88,219 |
| 196 | *Trogoderma granarium* | Beijing, China(Lab rearing) | 166.73 | 28 | 24 | 90,862 | 75,328 |
| 197 | *Trogoderma granarium* | Beijing, China(Lab rearing) | 159.7 | 24 | 21 | 77,036 | 64,876 |
| 198 | *Trogoderma granarium* | Beijing, China(Lab rearing) | 145.71 | 30 | 26 | 96,214 | 79,633 |
| 169 | *Bactrocera cucurbitae* | Beijing, China(Lab rearing) | 26.7 | 19.6 | 18 | 65,234 | 57,356 |
| 170 | *Bactrocera cucurbitae* | Beijing, China(Lab rearing) | 3.89 | 26 | 23 | 86,292 | 74,433 |
| 171 | *Bactrocera cucurbitae* | Beijing, China(Lab rearing) | 11.53 | 0.13 | 0.057 | 424 | 49 |
| 172 | *Bactrocera cucurbitae* | Beijing, China(Lab rearing) | 8.9 | 32 | 27 | 100,670 | 85,782 |
| 173 | *Bactrocera cucurbitae* | Beijing, China(Lab rearing) | 33.23 | 34 | 30 | 111,070 | 91,424 |
| 174 | *Bactrocera cucurbitae* | Beijing, China(Lab rearing) | 4.9 | 28 | 25 | 92,402 | 82,506 |
| 175 | *Bactrocera cucurbitae* | Beijing, China(Lab rearing) | 13.59 | 30 | 26 | 96,954 | 83,404 |
| 176 | *Bactrocera cucurbitae* | Beijing, China(Lab rearing) | 35.18 | 22 | 20 | 73,186 | 64,243 |
| 177 | *Bactrocera cucurbitae* | Beijing, China(Lab rearing) | 59.56 | 30 | 27 | 98,028 | 85,305 |
| 178 | *Bactrocera cucurbitae* | Beijing, China(Lab rearing) | 5.57 | 30 | 26 | 96,282 | 83,086 |
| 179 | *Bactrocera cucurbitae* | Beijing, China(Lab rearing) | 9.68 | 24 | 22 | 80,230 | 70,418 |
| 180 | *Bactrocera cucurbitae* | Beijing, China(Lab rearing) | 14.29 | 28 | 25 | 90,368 | 78,074 |
| 124 | *Solenopsis invicta* | Jinghong, Yunnan, China | 0.97 | 24 | 21 | 76,194 | 63,630 |
| 125 | *Solenopsis invicta* | Jinghong, Yunnan, China | 1.5 | 40 | 35 | 129,366 | 112,125 |
| 107 | *Dendroctonus pseudotsugae* | Taicang, Jiangsu, China | 13.35 | 24 | 20 | 75,246 | 63,105 |
| 108 | *Dendroctonus pseudotsugae* | Taicang, Jiangsu, China | 19.44 | 32 | 28 | 104,990 | 83,408 |
| 109 | *Dendroctonus pseudotsugae* | Taicang, Jiangsu, China | 9.8 | 30 | 27 | 100,354 | 88,254 |
| 110 | *Dendroctonus pseudotsugae* | Taicang, Jiangsu, China | 8.86 | 32 | 28 | 102,640 | 85,043 |
| 111 | *Dendroctonus pseudotsugae* | Taicang, Jiangsu, China | 14.49 | 26 | 23 | 85,200 | 75,105 |
| 72 | *Bactrocera dorsalis* | Beijing, China(Lab rearing) | 5.3 | 30 | 26 | 96,956 | 82,036 |
| 73 | *Bactrocera dorsalis* | Beijing, China(Lab rearing) | 5.19 | 26 | 23 | 85,204 | 72,917 |
| 74 | *Bactrocera dorsalis* | Beijing, China(Lab rearing) | 5.85 | 26 | 23 | 86,316 | 74,316 |
| 75 | *Bactrocera dorsalis* | Beijing, China(Lab rearing) | 3.38 | 26 | 22 | 83,080 | 72,095 |
| 76 | *Bactrocera dorsalis* | Beijing, China(Lab rearing) | 3.07 | 18.8 | 17 | 62,692 | 55,309 |
| 1 | *Leptinotarsa decemlineata* | Urumqi, the Xinjiang Uygur Autonomous Region, China | 670.47 | 38 | 33 | 123,296 | 102,809 |
| 2 | *Leptinotarsa decemlineata* | Urumqi, the Xinjiang Uygur Autonomous Region, China | 892.25 | 32 | 29 | 107,302 | 89,862 |
| 3 | *Leptinotarsa decemlineata* | Urumqi, the Xinjiang Uygur Autonomous Region, China | 35.93 | 34 | 30 | 112,934 | 95,863 |
| 4 | *Leptinotarsa decemlineata* | Urumqi, the Xinjiang Uygur Autonomous Region, China | 88.16 | 38 | 33 | 124,422 | 100,516 |
| 5 | *Leptinotarsa decemlineata* | Urumqi, the Xinjiang Uygur Autonomous Region, China | 9.69 | 30 | 27 | 98,342 | 85,244 |
| 6 | *Leptinotarsa decemlineata* | Urumqi, the Xinjiang Uygur Autonomous Region, China | 27.69 | 6.4 | 5.7 | 21,376 | 18,252 |
| 7 | *Leptinotarsa decemlineata* | Urumqi, the Xinjiang Uygur Autonomous Region, China | 48.74 | 26 | 23 | 86,750 | 73,021 |
| 8 | *Leptinotarsa decemlineata* | Urumqi, the Xinjiang Uygur Autonomous Region, China | 24.42 | 24 | 22 | 79,692 | 69,298 |
| 9 | *Leptinotarsa decemlineata* | Urumqi, the Xinjiang Uygur Autonomous Region, China | 76.78 | 28 | 25 | 92,714 | 80,016 |
| 16 | *Leptinotarsa decemlineata* | Suifenhe, Heilongjiang, China | 23.06 | 38 | 33 | 122,058 | 102,119 |
| 17 | *Leptinotarsa decemlineata* | Suifenhe, Heilongjiang, China | 24.67 | 30 | 25 | 96,470 | 76,947 |
| 18 | *Leptinotarsa decemlineata* | Suifenhe, Heilongjiang, China | 74.92 | 30 | 27 | 100096 | 82337 |
| 19 | *Leptinotarsa decemlineata* | Suifenhe, Heilongjiang, China | 74.31 | 36 | 32 | 119,348 | 99,630 |
| 20 | *Leptinotarsa decemlineata* | Mishan, Heilongjiang, China | 122.02 | 34 | 29 | 109,818 | 92,580 |
| 21 | *Leptinotarsa decemlineata* | Mishan, Heilongjiang, China | 365.71 | 24 | 22 | 79,868 | 68,195 |
| 22 | *Leptinotarsa decemlineata* | Mishan, Heilongjiang, China | 256.77 | 26 | 22 | 80,878 | 69,909 |
| 147 | *Leptinotarsa decemlineata* | Mishan, Heilongjiang, China | 43.99 | 28 | 25 | 92,594 | 77,303 |
| 202 | *Leptinotarsa decemlineata* | Mishan, Heilongjiang, China | 245.92 | 26 | 23 | 83,882 | 72,339 |
| 203 | *Leptinotarsa decemlineata* | Mishan, Heilongjiang, China | 203.97 | 28 | 25 | 93,658 | 80,481 |
| 27 | *Henosepilachna vigintioctopunctata* | Dongning, Heilongjiang, China | 80.23 | 28 | 25 | 93,612 | 79,631 |
| 28 | *Henosepilachna vigintioctopunctata* | Dongning, Heilongjiang, China | 117.05 | 42 | 38 | 140,648 | 120,691 |
| 29 | *Henosepilachna vigintioctopunctata* | Dongning, Heilongjiang, China | 69.52 | 34 | 30 | 112,460 | 95,827 |
| 30 | *Henosepilachna vigintioctopunctata* | Dongning, Heilongjiang, China | 35.95 | 18.6 | 17 | 61,872 | 51,118 |
| 31 | *Henosepilachna vigintioctopunctata* | Hulin, Heilongjiang, China | 93.87 | 28 | 25 | 90,406 | 79,031 |
| 32 | *Henosepilachna vigintioctopunctata* | Hulin, Heilongjiang, China | 88.95 | 32 | 29 | 105,464 | 89,573 |
| 33 | *Henosepilachna vigintioctopunctata* | Hulin, Heilongjiang, China | 128.1 | 28 | 25 | 91,454 | 78,455 |
| 34 | *Henosepilachna vigintioctopunctata* | Hulin, Heilongjiang, China | 126.27 | 24 | 22 | 79,250 | 69,404 |
| 35 | *Henosepilachna vigintioctopunctata* | Hulin, Heilongjiang, China | 156.36 | 28 | 25 | 90,936 | 79,575 |
| 58 | *Henosepilachna vigintioctopunctata* | Hulin, Heilongjiang, China | 211.32 | 32 | 28 | 105,228 | 86,584 |
| 59 | *Henosepilachna vigintioctopunctata* | Hulin, Heilongjiang, China | 572.32 | 28 | 25 | 93,512 | 78,906 |
| 60 | *Henosepilachna vigintioctopunctata* | Hulin, Heilongjiang, China | 59.22 | 15.6 | 14 | 52,126 | 44,679 |
| 61 | *Henosepilachna vigintioctopunctata* | Hulin, Heilongjiang, China | 36.9 | 26 | 23 | 83,838 | 70,926 |
| 62 | *Henosepilachna vigintioctopunctata* | Hulin, Heilongjiang, China | 223.75 | 32 | 28 | 103,718 | 87,873 |
| 113 | *Henosepilachna vigintioctopunctata* | Suifenhe, Heilongjiang, China | 31.26 | 34 | 29 | 109,096 | 90,783 |
| 114 | *Henosepilachna vigintioctopunctata* | Suifenhe, Heilongjiang, China | 12.2 | 24 | 21 | 77,896 | 65,920 |
| 115 | *Henosepilachna vigintioctopunctata* | Suifenhe, Heilongjiang, China | 35.91 | 54 | 47 | 175,474 | 147,458 |
| 116 | *Henosepilachna vigintioctopunctata* | Suifenhe, Heilongjiang, China | 29.17 | 19.4 | 18 | 64,688 | 55,777 |
| 117 | *Henosepilachna vigintioctopunctata* | Suifenhe, Heilongjiang, China | 7.48 | 30 | 26 | 95,840 | 83,284 |
| 99 | *Bactrocera tau* | Beijing, China(Lab rearing) | 19.67 | 17.6 | 16 | 58,458 | 49,831 |
| 100 | *Bactrocera tau* | Beijing, China(Lab rearing) | 13.28 | 28 | 25 | 90,384 | 77,388 |
| 101 | *Bactrocera tau* | Beijing, China(Lab rearing) | 8.86 | 22 | 20 | 73,464 | 62,468 |
| 102 | *Bactrocera tau* | Beijing, China(Lab rearing) | 7.59 | 30 | 26 | 94,530 | 81,460 |
| 103 | *Bactrocera tau* | Beijing, China(Lab rearing) | 19.48 | 30 | 25 | 93,796 | 79,437 |
| 104 | *Bactrocera tau* | Beijing, China(Lab rearing) | 0.11 | 28 | 25 | 91,064 | 77,110 |
| 105 | *Bactrocera tau* | Beijing, China(Lab rearing) | 9.87 | 32 | 28 | 103,358 | 85,488 |
| 106 | *Bactrocera tau* | Beijing, China(Lab rearing) | 69.18 | 32 | 29 | 107,058 | 92,016 |
| 165 | *Bactrocera tau* | Beijing, China(Lab rearing) | 67.04 | 30 | 26 | 97,446 | 82,548 |
| 166 | *Bactrocera tau* | Beijing, China(Lab rearing) | 19.55 | 32 | 28 | 101,180 | 87,621 |
| 167 | *Bactrocera tau* | Beijing, China(Lab rearing) | 11.17 | 26 | 22 | 81,050 | 68,684 |
| 168 | *Bactrocera tau* | Beijing, China(Lab rearing) | 43.24 | 24 | 21 | 74,934 | 64,568 |
| 187 | *Platypus parallelus* | Yangzhou,Jiangsu,China(Intercepted from the Solomon islands) | 1.29 | 36 | 32 | 118,686 | 100,122 |
| 188 | *Platypus parallelus* | Yangzhou,Jiangsu,China(Intercepted from the Solomon islands) | 1.77 | 22 | 20 | 73,014 | 63,471 |
| 189 | *Platypus parallelus* | Yangzhou,Jiangsu,China(Intercepted from the Solomon islands) | 2.3 | 30 | 27 | 98,414 | 83,773 |
| 118 | *Cydia pomonella* | Dongning, Heilongjiang, China | 11.55 | 26 | 23 | 84,282 | 71,440 |
| 119 | *Cydia pomonella* | Dongning, Heilongjiang, China | 4.18 | 28 | 25 | 91,814 | 78,652 |
| 120 | *Cydia pomonella* | Dongning, Heilongjiang, China | 8.72 | 26 | 22 | 83,478 | 69,046 |
| 121 | *Cydia pomonella* | Dongning, Heilongjiang, China | 5.46 | 32 | 27 | 101,484 | 81,648 |
| 11 | *Cydia pomonella* | Mudanjiang, Heilongjiang, China | 3.9 | 24 | 22 | 79,338 | 69,044 |
| 12 | *Cydia pomonella* | Mudanjiang, Heilongjiang, China | 13.22 | 24 | 20 | 74,196 | 64,095 |
| 13 | *Cydia pomonella* | Mudanjiang, Heilongjiang, China | 2.75 | 28 | 24 | 89,158 | 78,374 |
| 14 | *Cydia pomonella* | Mudanjiang, Heilongjiang, China | 16.34 | 32 | 28 | 103,688 | 87,260 |
| 15 | *Cydia pomonella* | Mudanjiang, Heilongjiang, China | 7.21 | 34 | 30 | 110,938 | 82,637 |
| 63 | *Cydia pomonella* | Urumqi, the Xinjiang Uygur Autonomous Region, China | 15.84 | 28 | 24 | 89,646 | 76,066 |
| 64 | *Cydia pomonella* | Urumqi, the Xinjiang Uygur Autonomous Region, China | 24.18 | 28 | 24 | 90,044 | 77,669 |
| 65 | *Cydia pomonella* | Urumqi, the Xinjiang Uygur Autonomous Region, China | 4.85 | 18.2 | 17 | 60,834 | 51,906 |
| 201 | *Cydia pomonella* | Urumqi, the Xinjiang Uygur Autonomous Region, China | 12.34 | 30 | 26 | 97,020 | 80,077 |
| 82 | *Cydia pomonella* | Urumqi, the Xinjiang Uygur Autonomous Region, China (Intercepted from Kazakhstan) | 2.62 | 32 | 27 | 101,214 | 80,334 |
| 83 | *Cydia pomonella* | Urumqi, the Xinjiang Uygur Autonomous Region, China (Intercepted from Kazakhstan) | 2.17 | 36 | 32 | 116,344 | 101,987 |
| 84 | *Cydia pomonella* | Urumqi, the Xinjiang Uygur Autonomous Region, China (Intercepted from Kazakhstan) | 5.49 | 32 | 28 | 104,646 | 85,988 |
| 85 | *Cydia pomonella* | Urumqi, the Xinjiang Uygur Autonomous Region, China (Intercepted from Kazakhstan) | 1.07 | 36 | 31 | 115,958 | 94,165 |
| 86 | *Cydia pomonella* | Urumqi, the Xinjiang Uygur Autonomous Region, China (Intercepted from Kazakhstan) | 3.14 | 32 | 28 | 105,382 | 86,733 |
| 129 | *Cydia pomonella* | Korla, the Xinjiang Uygur Autonomous Region, China | 5.31 | 22 | 20 | 73,484 | 63,733 |
| 130 | *Cydia pomonella* | Korla, the Xinjiang Uygur Autonomous Region, China | 6.33 | 30 | 26 | 94,500 | 80,533 |
| 131 | *Cydia pomonella* | Korla, the Xinjiang Uygur Autonomous Region, China | 5.19 | 30 | 26 | 97,716 | 82,687 |
| 132 | *Cydia pomonella* | Korla, the Xinjiang Uygur Autonomous Region, China | 2.84 | 20 | 18 | 66,398 | 56,300 |
| 133 | *Cydia pomonella* | Korla, the Xinjiang Uygur Autonomous Region, China | 5.3 | 28 | 25 | 91,478 | 77,508 |
| 66 | *Cydia pomonella* | Ili, the Xinjiang Uygur Autonomous Region, China | 6.86 | 42 | 37 | 137,196 | 117,395 |
| 126 | *Eriosoma lanigerum* | Menglian, Yunnan, China | 3.76 | 32 | 27 | 102,296 | 84,011 |
| 127 | *Eriosoma lanigerum* | Menglian, Yunnan, China | 4.3 | 30 | 26 | 95,052 | 79,658 |
| 128 | *Eriosoma lanigerum* | Menglian, Yunnan, China | 4.21 | 58 | 50 | 188,188 | 156,747 |
| 145 | *Eriosoma lanigerum* | Menglian, Yunnan, China | 1.88 | 24 | 22 | 78,618 | 68,570 |
| 157 | *Eriosoma lanigerum* | Menglian, Yunnan, China | 0.97 | 18.4 | 17 | 61,242 | 53,150 |
| 158 | *Eriosoma lanigerum* | Menglian, Yunnan, China | 0.42 | 26 | 23 | 83,436 | 71,607 |
| 159 | *Eriosoma lanigerum* | Menglian, Yunnan, China | 0.68 | 22 | 19 | 70,770 | 59,724 |
| 160 | *Eriosoma lanigerum* | Menglian, Yunnan, China | 0.74 | 30 | 27 | 100,088 | 83,948 |
| 136 | *Lymantria dispar* | Beijing, China(Lab rearing) | 64.38 | 28 | 25 | 89,762 | 78,769 |
| 137 | *Lymantria dispar* | Beijing, China(Lab rearing) | 50.97 | 16 | 15 | 53,134 | 46,642 |
| 184 | *Dysmicoccus neobrevipes* | Guangzhou, Guangdong, China | 41.5 | 30 | 26 | 96,352 | 84,724 |
| 185 | *Dysmicoccus neobrevipes* | Guangzhou, Guangdong, China | 54.42 | 36 | 32 | 119,364 | 99,674 |
| 186 | *Dysmicoccus neobrevipes* | Guangzhou, Guangdong, China | 70.17 | 30 | 26 | 94,100 | 83,187 |
| 25 | *Brontispa longissima* | Haikou, Hainan, China | 6.32 | 36 | 31 | 115,420 | 98,163 |
| 26 | *Brontispa longissima* | Haikou, Hainan, China | 8.1 | 28 | 24 | 87,624 | 76,870 |
| 57 | *Brontispa longissima* | Haikou, Hainan, China | 6.32 | 34 | 30 | 111,624 | 90,538 |
| 23 | *Opisina arenosella* | Haikou, Hainan, China | 53.17 | 30 | 26 | 96,272 | 82,046 |
| 24 | *Opisina arenosella* | Haikou, Hainan, China | 18.85 | 28 | 25 | 93,214 | 80,231 |
| 56 | *Opisina arenosella* | Haikou, Hainan, China | 17.81 | 28 | 25 | 91,630 | 80,694 |
| 134 | *Sitophilus zeamais* | Shanghai, China | 5.01 | 32 | 28 | 105,074 | 89,530 |
| 135 | *Sitophilus zeamais* | Shanghai, China | 5.72 | 22 | 18 | 67,132 | 58,226 |
| 204 | *Sitophilus zeamais* | Shanghai, China | 12.53 | 30 | 26 | 96,096 | 80,358 |
| 67 | *Ips typographus* | Taicang, Jiangsu, China | 3.97 | 36 | 32 | 118,542 | 97,859 |
| 68 | *Ips typographus* | Taicang, Jiangsu, China | 1.74 | 32 | 27 | 101,756 | 82,250 |
| 69 | *Ips typographus* | Taicang, Jiangsu, China | 0.83 | 32 | 29 | 105,272 | 92,676 |
| 70 | *Ips typographus* | Taicang, Jiangsu, China | 1.56 | 24 | 20 | 74,194 | 63,485 |
| 71 | *Ips typographus* | Taicang, Jiangsu, China | 1.17 | 32 | 28 | 103,474 | 87,222 |
| 77 | *Ips typographus* | Huangdao, Shandong, China (Intercepted from Czech) | 3.25 | 26 | 22 | 80,624 | 69,541 |
| 78 | *Ips typographus* | Huangdao, Shandong, China (Intercepted from Czech) | 2.62 | 30 | 26 | 94,072 | 81,640 |
| 79 | *Ips typographus* | Huangdao, Shandong, China (Intercepted from Czech) | 3.7 | 20 | 18 | 66,570 | 55,117 |
| 80 | *Ips typographus* | Huangdao, Shandong, China (Intercepted from Czech) | 2.97 | 38 | 34 | 127,444 | 108,081 |
| 81 | *Ips typographus* | Huangdao, Shandong, China (Intercepted from Czech) | 2.35 | 30 | 27 | 99,202 | 80,573 |
| 138 | *Ips typographus* | Suifenhe, Heilongjiang, China | 2.85 | 26 | 23 | 84,326 | 73,084 |
| 141 | *Ips typographus* | Suifenhe, Heilongjiang, China | 3.15 | 24 | 20 | 74,420 | 65,587 |
| 142 | *Ips typographus* | Suifenhe, Heilongjiang, China | 2.14 | 24 | 21 | 74,728 | 65,440 |
| 149 | *Ips typographus* | Suifenhe, Heilongjiang, China | 3.02 | 26 | 24 | 86,452 | 76,110 |
| 150 | *Ips typographus* | Suifenhe, Heilongjiang, China | 3.96 | 34 | 29 | 108,618 | 91,677 |
| 151 | *Ips typographus* | Suifenhe, Heilongjiang, China | 29.42 | 24 | 20 | 73,720 | 63,867 |
| 152 | *Ips typographus* | Suifenhe, Heilongjiang, China | 6.52 | 26 | 22 | 82,066 | 70,556 |
| 181 | *Carpomya vesuviana* | Ili, the Xinjiang Uygur Autonomous Region, China | 6.61 | 32 | 28 | 103,152 | 88,382 |
| 182 | *Carpomya vesuviana* | Ili, the Xinjiang Uygur Autonomous Region, China | 0.56 | 32 | 29 | 105,620 | 91,904 |
| 183 | *Carpomya vesuviana* | Ili, the Xinjiang Uygur Autonomous Region, China | 2.52 | 24 | 21 | 77,936 | 68,116 |
